# Impact of specialized mobility services on independent travel, economic outcomes, and well-being for people with low vision and blindness: an international survey

**DOI:** 10.1186/s12889-026-29083-5

**Published:** 2026-08-19

**Authors:** Maxime Bleau, Kopila Kafle, Min Wang, Soutongnoma Safiata Kaboré, Jorge Luis Cueva-Vargas, Yingzi Xiong, Joseph Paul Nemargut

**Affiliations:** 1 School of optometry, University of Montreal, 3744 rue Jean Brillant, Montreal, QC H3T 1P1 Canada; 2 Centre Interdisciplinaire de Recherche sur le Cerveau et l’Apprentissage, Montreal, QC Canada; 3 Centre de Recherche Interdisciplinaire en Réadaptation du Montréal métropolitain, Montreal, QC Canada; 4 Universidad Cesar Vallejo, Trujillo, La Libertad Peru; 5 Wilmer Eye Institute, Johns Hopkins University, Baltimore, MD USA; 6 Institut Nazareth et Louis-Braille, CISSS Montérégie-Centre, Longueuil, QC Canada; 7 Centre de réadaptation Lethbridge-Layton-Mackay du CIUSSS du Centre-Ouest de l’Île de Montréal, Montreal, QC Canada

**Keywords:** Vision rehabilitation, Visual impairment, Mobility, Orientation, Travel, Accessibility, Well-being, Blind, Low vision

## Abstract

**Background:**

Though independent mobility has a considerable impact on an individual’s quality of life, employability, and social participation, few studies have examined the impact of specialized mobility services for people with visual impairments (PVI) globally. This study examines the impact of these services on independent travel and how it relates to social determinants of health for adults with low vision and blindness.

**Methods:**

PVI 18 years of age or older were recruited via social media platforms or associations for the visually impaired to respond to online questionnaires translated in English, French, Spanish, and Mandarin. Survey 1 included questions related to place of residence, level of functional vision, travel habits, age, ethnicity, employment, and education. Survey 2 included questions related to the types of travel and mobility services received and perceived well-being.

**Results:**

Responses were received from 752 PVI in survey 1 and 584 PVI in survey 2 residing in 38 different countries. Only 35.6% of participants received specialized mobility services. Those who were more likely to have received services mentioned their vision is useless for navigation (*p*<.001), had an early diagnosis of visual impairment (< 14 years old; *p*<.05), and lived in urban areas (*p*<.001). The reasons for not receiving instruction varied significantly based on the region (*p*<.001), with the most common reason being the lack of availability. Those who received these services had higher perceived well-being (*p*<.001), economic success (*p*<.01), and level of education completed (*p*<.01) and ask for assistance less frequently (*p*<.05).

**Conclusions:**

Specialized mobility instruction is associated with higher independence and well-being of PVI around the world. These services should be made more accessible to promote independent travel amongst PVI and potentially reduce the burden on caregivers and society. Attention should be given to individuals living in rural areas, with a lower socioeconomic status, and a later onset of visual impairment, as many do not have access to these services.

**Supplementary Information:**

The online version contains supplementary material available at https://doi.org/10.1186/s12889-026-29083-5.

## Background

At least 2.2 billion people globally have a visual impairment [1], which is expected to increase across Global North countries as populations continue to age [2]. Hundreds of millions of adults currently live with an irreversible impairment, such as age-related macular degeneration, glaucoma, or diabetic retinopathy that is not improved by current ophthalmologic or optometric interventions [3]. In addition to the impacts on physical health and functioning, having a visual impairment may impact an individual’s place in society, influencing several social determinants of health. Social determinants of health are the non-medical conditions in which people are born, grow, work, live, and age that influence their health outcomes [4]. Individuals with visual impairments often encounter substantial barriers in accessing higher education [5–7] and employment [8–10], which reduces their income potential [11].

Much of the research in this field has been focused on the treatment and prevention of visual impairment. Though vision rehabilitation has been demonstrated to improve the quality of life and well-being of people with visual impairments (PVI) [12–16], it has received relatively less attention. Additionally, the ability to read or perform daily activities at home are often the primary metrics analyzed to determine the effectiveness of vision rehabilitation [17–19]. Though the impact of visual impairments on transportation, shopping, and leisure activities have been studied extensively [20–26], the impact of rehabilitation to address these needs through intervention is less well understood.

### O&M services

Orientation and mobility (O&M) instruction is a branch of vision rehabilitation that serves to improve the mobility and travel outcomes of PVI [27, 28]. Since 2000, many professionals in this specialized field are certified orientation and mobility specialists (COMS) after completing training in accredited universities and passing a standardized exam [29]. COMS provide training that empowers individuals with blindness or low vision to travel safely, efficiently, and as independently as possible in home, school, and community environments [27]. COMS train PVI in the use of sensory substitution tools, such as white canes and guide dogs, to be able to detect objects using tactile or kinesthetic senses, or tactile maps to develop mental maps without vision [30, 31]. Also, COMS enhance auditory skills for navigation, using echolocation, sound localization, amongst other techniques [25, 31, 32]. There are many countries, where, to the best of our knowledge, these types of specialized travel services for the visually impaired are severely lacking or non-existent [33].

### Impact of O&M

Individuals with acquired visual impairments, regardless of their age group, may be more likely to experience anxiety [34] and/or depressive symptoms due to limited physical activity and sedentary behaviors [35–37]. Fortunately, O&M services have been shown to reverse some of these trends amongst PVI by improving their psychological and physical well-being [13, 38, 39]. Additionally, recent standardized assessment tools have demonstrated the effectiveness of O&M services for certain interventions [40–42].

In the educational system, O&M services are often offered to visually impaired students by COMS through the expanded core curriculum during primary and secondary education [28, 43, 44]. Though PVI are still often underemployed [45], longitudinal studies have demonstrated that O&M service provision may enhance both educational and employment outcomes for PVI [46, 47].

Not only do these services improve the mobility and employment outcomes as well as the quality of life of PVI, but recent evidence has shown a possible economic benefit for society: Australian researchers found, using the willingness to pay model, O&M programs generate benefits for clients in excess of the cost of providing the services, and thus is deemed a socially efficient investment [48]. Despite the numerous positive impacts reported by PVI and documented by researchers for O&M services [49, 50], a global study of the associated impacts of these services to well-being, independent travel, education and employment has not been conducted.

### Objectives

As raised by the Lancet Global Commission on Global Eye Health, insufficient systematic data remains a persistent issue in raising the profile of vision rehabilitation and understanding the extent of the problem [33]. Thus, our study aims to investigate the access to and associated impact of O&M services on an international level, based on the responses of people living with low vision or blindness to an online questionnaire. This study on O&M services examines the following 4 research questions:

1. How are O&M services correlated with the level of independent travel?
2. How are O&M services correlated with perceived well-being?
3. Are O&M services correlated with educational and employment outcomes?
4. How do O&M services differ in different regions of the world?

## Methods

This study is part of a larger study entitled *“The road to independent navigation: an international survey on orientation skills of individuals living with visual impairments.”* Therefore, parts of the methodology and collected data have been reported by our laboratory elsewhere [12].

### Inclusion criteria and ethical considerations

All data was gathered through two online surveys, published internationally on different social media channels and shared through specialized associations for people with visual impairments. All surveys were assessed prior to deployment to ensure full accessibility to commonly used screen reading and magnification software, such as Zoomtext and JAWS. However, considering potential technological barriers for the visually impaired population responding to online surveys (i.e. access to accessible software and computer training), associations of the visually impaired were encouraged to provide technical support, such as accessible computers, internet, software, and assistance for those that were not able to complete the survey independently at their desired location. Additionally, a member of the research team read the surveys over the phone or while using videoconferencing software for those that wished to participate but lacked access to the surveys.

Prior to responding to the actual survey questions, participants received information about the research and its purpose via an information sheet and were informed that responding to the survey would be considered as their given consent to participate in the study. To be retained as participants, respondents had to be at least 18 years old and self-identify as having a visual impairment, defined as an uncorrectable visual condition. The study was conducted in accordance with the declaration of Helsinki. The ethics approval was obtained from the Comité d’éthique à la recherche Clinique (CERC) of the Université de Montréal (2023–5070) on November 24th, 2023. Though associations of the visually impaired provided critical support for recruitment, it was not appropriate to involve them or the public in the design, or conduct, or reporting, or dissemination plans of our research due to the specialized nature of the services studied and the lack of such services in many of the areas studied.

### Survey description

Data was collected using two online surveys administered through LimeSurvey between December 2023 and September 2024. The survey was published in English, French, Spanish, and Mandarin; all versions of the survey were verified by native speakers with experience in vision rehabilitation. The questionnaires were restricted to these four languages due to potential inaccuracies of both translated questions as well as responses of participants to open-ended questions that could not be validated by native speakers with experience in vision rehabilitation on the research team. The survey was divided into two parts, and participants received the link to the second survey after having completed the first one. The full English version of the questions analyzed in the 2 surveys is available in Supplementary file S1.

Survey 1 was divided into 4 sections. Section  1 was comprised of 18 to 21 questions about sociodemographic factors such as age, gender, area of living, visual condition, age at diagnosis, degree of functional vision, and use of mobility aids. Section  2 was comprised of 19 to 27 questions about traveling habits, such as mobility aids, how frequently they travel outside the home (independently vs. accompanied, or in familiar vs. unfamiliar areas), and their level of confidence when traveling. Section  3 was comprised of 7 questions and served as a questionnaire to score the cognitive mapping skills on a scale to 100% according to their confidence in various situations. Finally, Sect.  4 was comprised of 4 to 25 questions about participants’ experience with tactile and visual maps which was reported previously from our laboratory [12] and is not included in the present manuscript.

Survey 2 was divided into 4 sections. Section  1 was comprised of 2 to 17 questions about the reception of specialized mobility training such as O&M services, how often they need them and what tools and/or strategies they learned. Section  2 was comprised of 10 to 15 questions about the various orientation strategies they use when travelling and how frequently they need assistance during travel. Section  3 was comprised of 6 questions, in which participants were given scenarios about wayfinding in indoor and outdoor environments, explaining their strategies and rating their confidence in both types of environments. Finally, Sect.  4 was a questionnaire based on the OMO tool part B [51], with ratings of participants’ general well-being, based on engagement in activities, personal connections, orientation abilities, life space and self-determination.

### Data filtering

Results from the survey were exported to Microsoft Excel, where incomplete submissions and duplicates were removed, with an expected attrition rate of around 50% for both surveys, estimated according to the web-based nature of the survey, its duration (over 30 min according to prior testing with PVI), and factors related to the added complexity of visual impairments and other disabilities such as platform accessibility, software or assistive technology issues, technological problems during participation, web navigation skills, and memory load when (re)reading questions and related multiple choices [52, 53].

### Statistical analysis

Analyses were performed in SPSS version 29 and JASP [54] and included non-parametric tests. Statistical tests were performed on different sets of selected variables of interest (see Table 1) and followed the same procedures as previously reported by the laboratory [12]. First, associations between social determinants of health, travel habits, well-being, and O&M services were assessed using: Mann-Whitney U, Chi-square, and Spearman Rho correlation tests, depending on the tested variables. Since analyses involved multiple tests, all p-values were adjusted using the Benjamini-Hochberg False Discovery Rate (FDR) correction. Finally, for multiple-response data (e.g., participants could select more than one option), Cochran’s Q tests were performed to assess if options were equally chosen. Significant results were followed by pairwise post-hoc Dunn’s multiple comparisons tests (with Bonferroni corrections) to identify differences between options. For each statistical test, if there was missing data for one or more respondents, their entry was excluded from that test.

**Table 1:** Variables used in the analysis, their data type, and corresponding survey questions, adapted from Bleau et al. [12]

Category	Variable	Data type	# Question
Socio demographics	Age	Continuous	S1, Q1.1
	Gender	Categorical	S1, Q1.2
	Country of residence	Categorical	S1, Q1.3
	Living area type	Categorical	S1, Q1.11
	Cause of VI	Categorical	S1, Q1.5
	If VI is or was gradual (progressive visual loss)	Binary	S1, Q1.7
	Age at VI diagnosis	Continuous	S1, Q1.6
	Utility of vision	Ordinal (4 levels)	S1, Q1.9
	Age at onset of blindness (if no usable vision)	Continuous	S1, Q1.10
	Highest level of education completed	Ordinal (7 levels)	S1, Q1.4
	Economic status	Ordinal (3 levels)	S1, Q1.12
Mobility Services	Title of professional who provided services	Categorical	S2, Q1.2
	How do they proceed to get mobility services	Categorical	S2, Q1.3
	Age at first mobility lessons	Discrete	S2, Q1.4
	Time since last lesson	Ordinal (5 levels)	S2, Q1.5
	Frequency of mobility lessons	Ordinal (5 levels)	S2, Q1.6
	Reason why they did not receive mobility services	Categorical	S2, Q1.7
Mobility & independence	How frequently they travel independently	Ordinal (5 levels)	S1, Q2.1
	How frequently they travel accompanied	Ordinal (5 levels)	S1, Q2.2
	For how many reasons do they travel	Discrete (0 to 7)	S1, Q2.3
	How accessible they perceive their environment	Ordinal (4 levels)	S1, Q2.4
	How frequently they travel in familiar areas	Ordinal (5 levels)	S1, Q2.5
	How frequently they travel in unfamiliar areas	Ordinal (5 levels)	S1, Q2.6
	Confidence when travelling in unfamiliar areas	Ordinal (6 levels)	S1, Q2.7
	Confidence when travelling in outdoor areas	Ordinal (10 levels)	S2, Q3.1
	Confidence when travelling in indoor areas	Ordinal (10 levels)	S2, Q3.2
	How frequently they require help during travels	Ordinal (5 levels)	S2, Q2.1
	Cognitive mapping ability score	Continuous	S1, Q3.1-6
Well-being	Highest level of education completed	Ordinal (7 levels)	S1, Q1.4
	Economic status	Ordinal (3 levels)	S1, Q1.12
	Well-being (OMO) score	Continuous	S2, Q4.1-5

The variables are classified according to survey and section (supplementary file S1.1). S1, survey 1; S2, survey 2; Q, question

Finally, to confirm the association between mobility services and social determinants of health, travel habits, and well-being, when accounting for confounding factors, a series of multiple linear regressions were performed. For these analyses, each outcome variable was entered separately as the dependent variable, while mobility services served as the predictor of interest. All models also included age, gender, highest education level, economic status, level of functional vision, age at VI diagnosis, WHO region and area type (rural, suburban, urban) as covariates, except for the education and economic status models, where the corresponding variable was omitted from the predictor set. Categorical predictors were entered as factors and dummy coded automatically by the statistical software. Regression coefficients (B), t statistics, p values, and adjusted coefficients of determination (adjusted R²) were extracted for interpretation.

## Results

In total, we received 1437 entries in survey 1 and 845 entries in survey 2. 595 incomplete responses and 90 duplicates were filtered out from survey 1; and 230 incomplete responses and 31 duplicates were filtered out from survey 2. As a result, 752 PVI (410 male, 341 female, 1 N/A, mean age = 36.44 y, range 18–86 y; 47.67% attrition rate from a total of 1437 entries) completed responses were provided for the first survey, which investigated demographic factors, life and travel habits, cognitive mapping skills, and the use of tactile maps. Survey 2, which investigated specialized mobility training, well-being outcomes (as defined by the OMO tool part B) [42], and confidence in indoor and outdoor environments, totaled 584 completed entries. Out of these entries, 510 corresponded to respondents in survey 1 (32.18% attrition rate from survey 1 to survey 2). On average, survey 1 took 30.33 min to complete and survey 2, 18.74 min. Only the completed entries from Survey 2 (with corresponding survey 1 entries or not) were analyzed for this study. Therefore, the final sample counted 584 individuals (293 male, 217 female, 74 N/A, mean age = 34.39 y, range 18–86 y). The total sample was used for tests in which only variables from survey 2 were analyzed and a subsample of 510 individuals for those in which variables from both surveys were jointly analyzed.

### Demographics

**Fig. 1:**
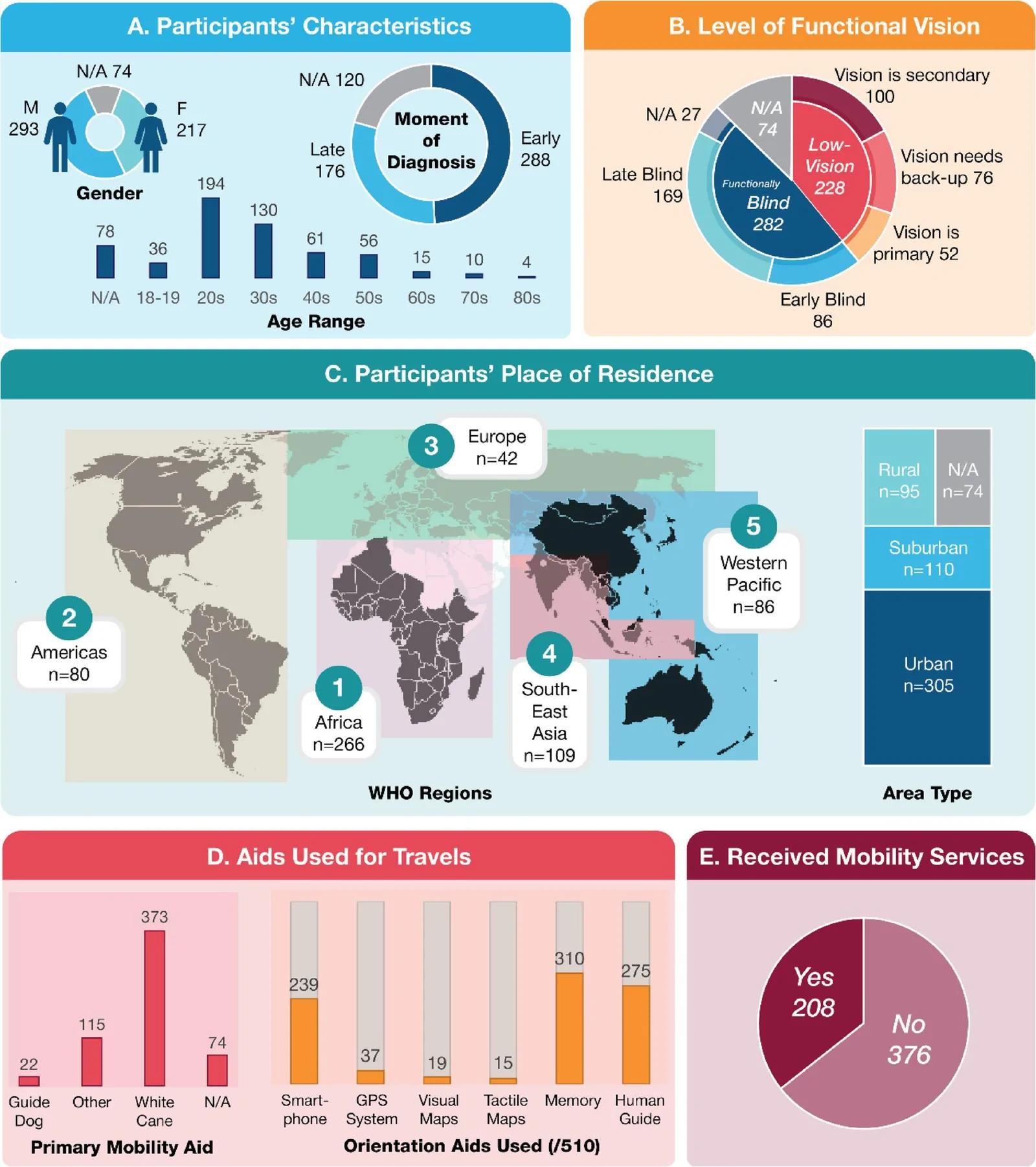
Sociodemographic profiles of participants. **A** Gender, age category, moment when the visual condition was diagnosed, categorized according to two age categories: early diagnosis [0–14 years], and late diagnosis [15 years and over]. **B** Level of functional vision (including early blind [blind before six years] and late blind [blind after six years]; from VROOM tool). **C** Place of residence according to the WHO world regions (from https://www.who.int/countries) and area type (urban, suburban, or rural). **D** Mobility and orientation aids used for travel, **E** Access to specialized mobility services. F, females; M, males; N/A, no answer

Participants were from 38 different countries, including five WHO regions; totaling 80 participants from the Americas (13.70%), 42 from Europe (7.19%), 266 from Africa (45.55%), 109 from South-East Asia (18.66%); 86 from the Western Pacific region (14.73%); and one from the Eastern Mediterranean region (0.17%, from Pakistan). Most participants declared themselves living in urban areas (*n* = 305, 52.23%), while the others lived either in suburban (*n* = 110, 18.84%) or rural (*n* = 95, 16.27%) areas. In general, most participants received their terminal degree from an undergraduate university program (*n* = 185, 31.68%), or from high school (*n* = 103, 17.64%) and were from lower (*n* = 311, 53.25%) or middle (*n* = 196, 33.56%) economic classes (in the context of their region). The VROOM tool [55] classification of visual functions was used to describe participants’ level of functional vision (e.g., how vision is used during navigation) and to categorize them as either functionally blind (vision useless for navigation) or having low-vision (vision either secondary, needing back-up or primary for navigation). Following this classification, 282 (48.29%) were identified as functionally blind and 228 (39.04%) as having low-vision (see Fig. 1B), For those who identified as blind, the mean age at blindness onset was 16.15 years, totaling 86 early blind (EB; blind before six years old) and 169 late blind participants (LB; blind after six years old). Figure 1 illustrates the sociodemographic profiles of all participants.

### Specialized mobility service provision

Out of the 584 participants who answered the second survey, only 208 (35.62%) had received mobility services. For those that had, they received it from an O&M instructor (*n* = 105, 50.48%), teacher (*n* = 60, 28.85%), or locomotion instructor (*n* = 25, 12.02%). They typically received these services from a school (*n* = 83, 39.90%), rehabilitation center (*n* = 34, 16.35%), or a non-governmental organization (*n* = 36, 17.31%). On average, they received their first lesson at 18.28 ± 13.14 years old, and they typically received their last lesson more than 5 years prior to responding to the survey (*n* = 123, 59.13%) or between 1 and 5 years prior (*n* = 38, 18.27%).

The likelihood of an individual receiving mobility services was significantly associated with several factors. Individuals living in rural areas were less likely to receive these services than those living in urban or suburban areas (X^2^(2, 510) = 16.201, *p* < .001); those whose VI was diagnosed in adulthood were also less likely to receive services compared to those diagnosed in their childhood (X^2^(2, 464) = 7.620, *p* = .022); those with either central or a peripheral visual loss were less likely to access services than those with generalized field visual loss affecting both central and peripheral fields (X^2^(2, 510) = 9.742, *p* = .008). For functionally blind participants, those with a later blindness onset were less likely to receive than those with an early onset (X^2^(2, 255) = 3.960, *p* = .047); similarly, those diagnosed in adulthood or late adulthood were less likely to receive than those who were diagnosed in childhood (X^2^(2, 255) = 9.048, *p* = .011).

As for the different reasons why the other 376 (64.38%) participants did not receive mobility services, a significant multinomial test (X^2^(4, *n* = 375) = 216.587, *p*<.001) revealed that some reasons were more common than others: the lack of availability (*n* = 174, 46.28%) was the most common, followed by the lack of interest (*n* = 99, 26.33%) while the lack of need (*n* = 56, 14.89%), or readiness (*n* = 30, 7.98%) were less common.

### Impact of specialized mobility services

We first examined the relationship between the provision of specialized mobility services and the different mobility measures assessed throughout the survey. Services were not related to the frequency of independent travels (3.583 vs. 3.393, U = 27860, *p* = .127, p_(FDR)_ = 0.200 *r* = .077), accompanied travels (2.642 vs. 2.567, U = 28395, *p* = .220, p_(FDR)_ = 0.269, *r* = .06), travels in unfamiliar areas (2.230 vs. 2.204, U = 29274, *p* = .430, p_(FDR)_ = 0.473, *r* = .031), confidence when traveling in unfamiliar areas (2.684 vs. 2.824, U = 27970, *p* = .136, p_(FDR)_ = 0.187, *r* = .074), or how accessible the participants viewed their environment (U =, *p*=.08, p_(FDR)_ = 0.147, *r* = .085). However, mobility services were related to better cognitive mapping skills (U = 25713.5, *p* = .005, p_(FDR)_ = 0.018, *r* = .149), more frequent travels in familiar areas (3.647 vs. 3.353, U = 25645.5, *p* = .003, p_(FDR)_ = 0.017, *r* = .151), a higher number of reasons for traveling (U = 22743.5, *p* < .001, p_(FDR)_ < 0.001, *r* = .247), and lesser needs for assistance while traveling (U = 22743.5, *p* = .019, p_(FDR)_ = 0.052, *r* = -.106).

Specialized mobility services also were differently linked to the frequency of independent travel in blind participants compared to low vision participants (F(1,506) = 7.889, *p*=.005, p₍_FDR_₎=0.028). Indeed, simple main effects show that these services were more impactful for blind (F(1) = 12.230, *p*<.001) than low vision participants (F(1) = 0.563, *p*=.453). Non-significant trends were also noted in their confidence when traveling in unfamiliar areas (F(1,506) = 5.635, *p*=.018, p₍_FDR_₎=0.066), where blind participants (F(1) = 9.155, *p*=.003) were more impacted than low vision participants (F(1) = 0.332, *p*=.565); and in their number of reasons to travel (F(1,506) = 4.538, *p*=.034, p₍_FDR_₎=0.094) where blind participants were, again, more impacted (F(1) = 27.364, *p*<.001) compared to low vision participants (F(1) = 2.456, *p*=.118).

**Fig. 2:**
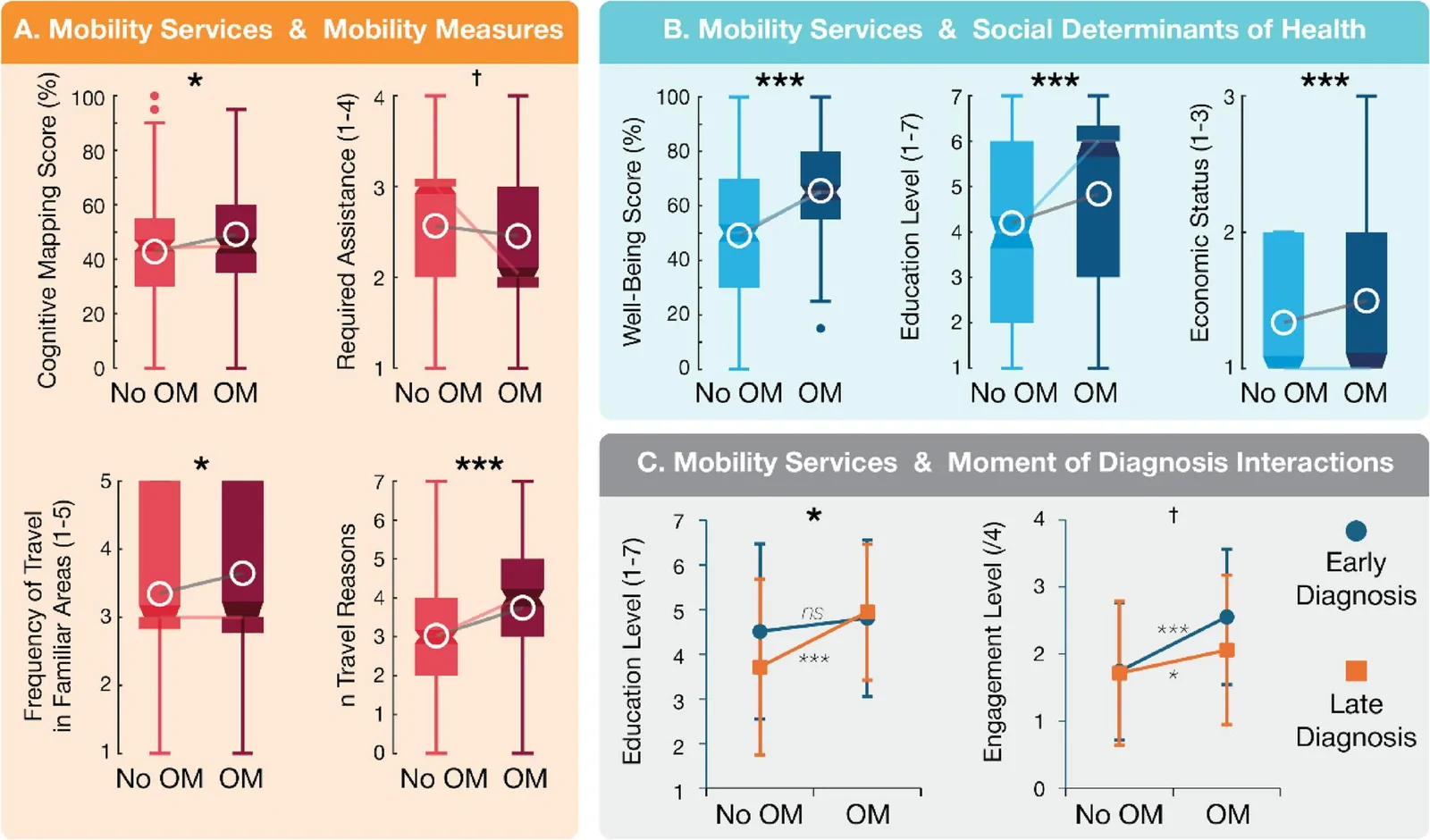
Relationships between the provision of specialized mobility services, mobility measures and social determinants of health in all participants (blind and low vision included). **A** Box plots displaying that the provision of mobility services was linked to cognitive mapping scores (continuous, 1-100%), the frequency required assistance during their travels (ordinal scale, 1-4), the frequency of travel in familiar areas (ordinal scale, 1-4), and the number of reasons to travel (discrete, 0-7). **B** Box plots displaying that the provision of mobility services was linked to well-being scores (continuous, 1-100%) as defined by the OMO tool, education levels (ordinal scale, 1-7), and economic status (ordinal scale, 1-3) **C** Error bar plots displaying how the relationships between the provision of mobility services and education level and engagement level (OMO tool, question 1) differ between participants with early and late diagnosis. The well-being score (0–100%) reflects the quality, or level, of engagement in activities, personal connections, orientation capacities, and life space, while the cognitive mapping skills score (0-100%) reflects participants’ ability and confidence to learn the layout of new environments. Most plots are on ordinal scales (more information on the ordinal scales in Supplementary files 1 and 2). The box and whiskers plots (**A**,**B**) also display the means (white circles), and the lines between boxes represent the difference between the OM and No OM sub-groups (dark lines = difference between means; light lines = difference between medians). The error bar plot also displays the average scores to facilitate comparisons between groups and subgroups. †, p<.10 (non-significant trend); *, p<.05; **, p<.01; ***, p<.001. No OM, without mobility services; OM, with mobility services

Specialized mobility services were related to higher levels of education (U = 24565.5, *p* < .001, *r* = .187) and economic success (U = 25693.5, *p* < .001, *r* = .149), as well as higher well-being scores (U = 23281.5, *p* < .001, *r* = .406) assessed with the OMO tool (see Fig. 2). More precisely, specialized mobility services were linked to all measures of the OMO tool; this included measures like engagement (U = 25738, *p* < .001, p_(FDR)_ < 0.001, *r* = .339), connections (U = 28680.5, *p* < .001, p_(FDR)_ < 0.001, *r* = .263), lifespace (U = 25913, *p* < .001, p_(FDR)_ < 0.001, *r* = .334), orientation (U = 27369.5, *p* < .001, p_(FDR)_ < 0.001, *r* = .297), and self-determination (U = 27180, *p* < .001, p_(FDR)_ < 0.001, *r* = .302).

**Fig. 3:**
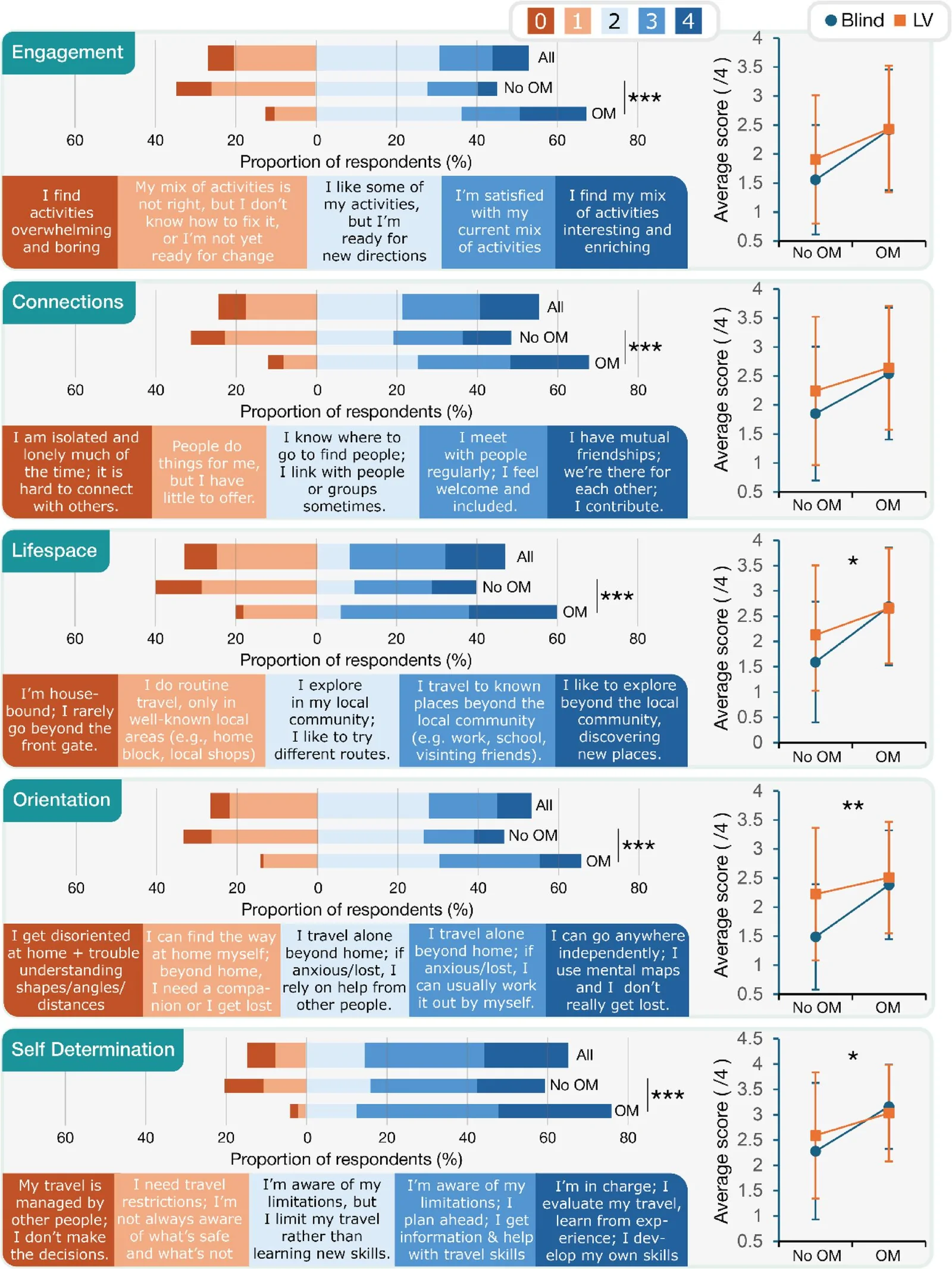
Participants’ responses to every question in the OMO tool and differences in response between blind and low-vision participants. The OMO tool assesses the quality, or level of five life spheres (engagement in activities, personal connections, orientation capacities, life space, and self-determination). (Left) Diverging bar plots represent proportion of respondents for each answer (5 levels per question). Answer options were rated on an ordinal scale ranging from 0 to 4 (lowest score to highest score) and are displayed with colors ranging from dark red (lowest, most negative) to dark blue (highest, most positive). (Right) Error bar plots represent the differences between the average scores of participants who received (OM) or did not receive (No OM) mobility services in both participants with blindness (blue) and low vision (orange). Average scores are all on the same ordinal scale shown on the left side and intermediate values are displayed for illustrative purposes. *, p<.05; **, p<.01; ***, p<.001. No OM, without mobility services; OM, with mobility services

Mobility services were also differently linked to well-being scores in low vision and blind respondents (F(1,506) = 7.786, *p*=.005, p_(FDR)_=0.015): services were more positively linked to well-being in blind participants (F(1, 281) = 73.988, *p*<.001; t(282) = 8.602, cohen’s d = 1.036, pbonf<0.001) compared to low vision participants (F(1, 227) = 12.267, *p*<.001; t(228) = 3.502, cohen’s d = 0.509 pbonf=0.003); this interaction was mostly true in life space (F(1,506) = 5.925, *p*=.015), orientation (F(1,506) = 10.512, *p*=.001), and self-determination spheres (F(1,506) = 4.036, *p*=.045). Blind and low vision participants were not differently impacted by mobility services in the other well-being measures, including education (F(1,506) = 0.719, *p*=.397, p_(FDR)_=0.596) and economic status (F(1,506) = 0.002, *p*=.962, p_(FDR)_=0.962). See Fig. 3 for additional information.

#### Mobility service impacts according to moment of VI diagnosis

We divided participants into two subgroups according to the moment of their VI diagnosis (early: ≤14 yo; late: 15 + yo). A significant chi square test (X^2^(1, *n* = 464) = 10.920, *p* < .001) revealed that participants with early VI diagnosis were more likely to receive mobility services (early diagnosis: 126/288, 43.75%; late diagnosis 50/176, 28.41%). Subsequently, ANOVAs revealed only one significant interaction according to which mobility services impact education these subgroups differently (F(1,460) = 4.720, *p*=.030, p₍_FDR_₎=0.09): indeed, O&M services were linked to increased levels of education for those with late diagnosis (F(1) = 15.466, *p*<.001), but not for those with early diagnosis (F(1) = 3.181, *p*=.075). This interaction was also mostly true for blind participants. Indeed, while EB (< 6 yo) had higher education levels than LB (6 + yo; U = 15644.5, *p*=.001, *r* = .22, p₍_FDR_₎=0.003), mobility services were more related to higher education levels in LB compared to EB (F(1,251) = 10.861, *p*=.001, p₍_FDR_₎=0.003; LB (F(1) = 22.959, *p*<.001; EB (F(1) = 0.315, *p*=.575).

Finally, looking closely at the questions in the OMO tool, interactions between age at diagnosis and O&M services revealed that individuals with early VI diagnosis may be impacted differently in the engagement sphere (F(1,506) = 5.013, *p*=.041, p₍_FDR_₎=0.205): O&M services seem linked to higher engagement levels for those with early diagnosis (F(1) = 49.377, *p*<.001) compared to those with late diagnosis (F(1) = 3.949, *p*=.047; see Fig. 2C).

#### Mobility services impact according to the moment of provision

The timing between VI diagnosis and first mobility service provision did not correlate with any mobility- or well-being-related measures. However, when examining subgroups defined by age at diagnosis, distinct patterns emerged.

For participants with a late diagnosis, the timing of mobility services correlated with higher perceived environmental accessibility, although this association did not survive FDR correction (rho=-0.323, *p*=.022, p_(FDR)_=0.242). In those with an early VI diagnosis, mobility services timing was significantly associated with travel frequency in familiar areas (rho = 0.265, *p*=.003, p_(FDR)_=0.033), indicating that participants who received mobility services later in life tended to travel more frequently in familiar environments. Among low-vision participants no significant correlations were found, except in the late-diagnosis subgroup: later mobility services were related to needing assistance more frequently when traveling (rho=0.530, *p*=.005, p_(FDR)_=0.055).

In blind individuals, a similar divergence appeared between EB and LB participants. For LB, the delay between first mobility lessons and age at VI diagnosis did not correlate with any measures, but when mobility services were referenced to age at onset of blindness, several relationships emerged. Earlier provision of mobility services in LB was associated with perceiving the environment as more accessible (rho=-0.657, *p*<.001, p_(FDR)_=0.011), traveling more frequently when accompanied (rho=-0.547, *p*=.008, p_(FDR)_=0.044), and, to a lesser extent, with higher confidence when traveling in unfamiliar areas (rho=-0.470, *p*=.027, p_(FDR)_=0.099). For EB, earlier provision of O&M services relative to diagnosis was linked to higher confidence when traveling both indoors (rho=-0.345, *p*<.001, p_(FDR)_=0.011) and outdoors (rho=-0.258, *p*=.014, p_(FDR)_=0.051). Later mobility services provision was also associated with more frequent travel in familiar areas (rho=0.340, *p*=.001, p_(FDR)_=0.006). When considering the timing of O&M services relative to the onset of blindness, no significant correlations emerged.

### Regional differences

Finally, we investigated inter-regional differences in the provision of mobility services and how differently these services were linked to social determinants of health and mobility measures across the different WHO regions. We found that the level of service provision differed according to the region where participants reside (X^2^(4, 583) = 83.625, *p* < .001). Indeed, compared to the general proportion in the survey (35.62% access), PVI in the Americas (62.50% access) and Europe (69.05% access) are more likely to receive services, while those in the South-East Asia (11.93% access) and Western Pacific (18.60% access) regions are less likely to receive services; Africa was not different from the general proportion (37.22% access). Proportions of PVI receiving services in the Americas and Europe were higher than those in Africa, South-East-Asia and Western Pacific regions. Proportions in Africa were also higher than in South-East Asia and Western Pacific regions (Supplementary file S1.2).

**Fig. 4:**
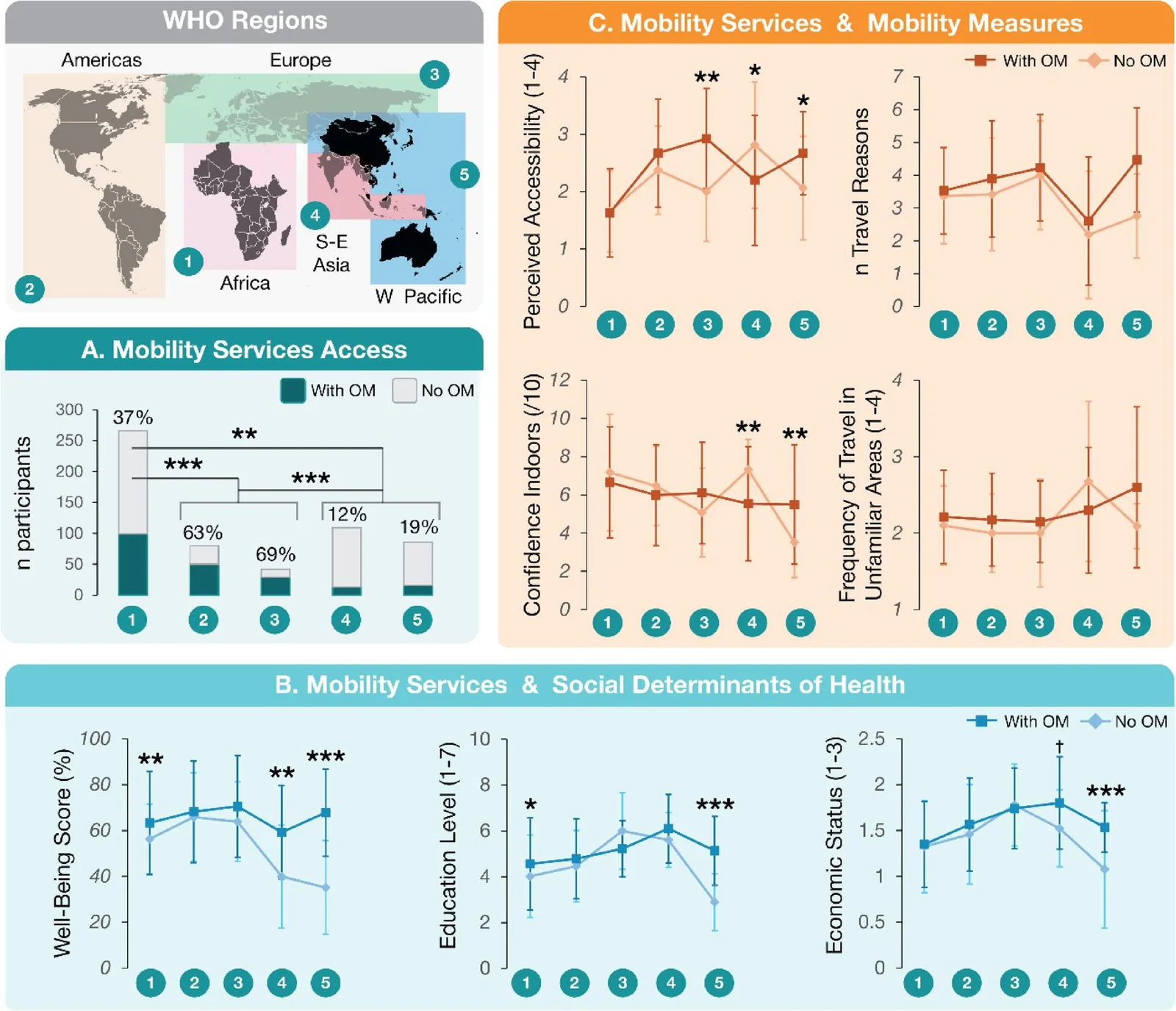
Regional differences in specialized mobility services impact. **A** Proportions of participants with and without access to mobility services across the five WHO regions. **B** Social determinants of health measures differ between participants with and without access to mobility services depending on WHO regions. **C** Mobility measures differ between participants with and without access to mobility services depending on WHO regions. The well-being score (0–100%) reflects the quality, or level, of engagement in activities, personal connections, orientation capacities, and life space. Mean ratings are on ordinal scales (i.e., 1 to 5, where each number represents a categorical, discrete level) and intermediate values are displayed for illustrative purposes (more information on the ordinal scales in Supplementary files S1.2). †, *p*<.10 (non-significant trend); *, *p*<.05; **, *p*<.01; ***, *p*<.001; S-E Asia, South-East Asia; W Pacific, Western Pacific

The general impact of O&M services also significantly varied across the world regions, as revealed by significant interactions of these factors on education levels (F(4,500) = 3.886, *p*=.004, p_(FDR)_=0.006), economic status (F(4,500) = 2.555, *p*=.038, p_(FDR)_=0.038) and overall well-being scores (F(4,573) = 5.855, *p*<.001, p_(FDR)_=0.003). O&M services were mostly linked to higher education in Africa (F = 5.570, *p*=.019) and the Western Pacific (F = 20.217, *p*<.001); to higher economic success in Western Pacific (F = 11.388, *p*<.001) and South-East Asia (F = 2.981, *p*=.085); and to well-being in Africa (F = 7.616, *p*=.006), South-East Asia (F = 10.597, *p*=.001) and Western Pacific (F = 34.600, *p*<.001). See Fig. 4 for additional information.

As for mobility measures, inter-regional variations in O&M services impacts were found in: (1) the perceived accessibility of the environment (F(4,500) = 4.737, *p*<.001, p_(FDR)_=0.011) where greater positive impacts were found in Europe (F = 8.017, *p*=.005), and Western Pacific (F = 6.183, *p*=.013) along with a negative effect in Southeast Asia (negative effect, F = 4.547, *p*=.033); (2) confidence when travelling indoors (F(4,573) = 4.198, *p*=.002, p_(FDR)_=0.011), where a positive effect was found in the Western Pacific (F = 7.696, *p*=.006) and a negative effect, in Southeast Asia (negative effect, F = 5.464, *p*=.002). To a lesser extent, interactions were also found in the number of travel reasons (F(4,500) = 2.537, *p*=.039, p_(FDR)_=0.143) with a main effect of services only in the Western Pacific region (F = 14.770, *p*<.001); and the frequency of travels in unfamiliar areas (F(4,500) = 2.427, *p*=.047, p_(FDR)_=0.129), again with a main effect only in the Western Pacific region (F = 7.598, *p*=.006).

The reasons for not receiving these services also differed according to regions (X^2^(16, *n* = 375) = 117.095, *p* < .001). In the Western Pacific, lack of availability was prominent (57/70, 81.43% vs. 46.4% in the general population). In South-East Asia there were lesser needs (30/96, 31.25% vs. 14.93% in the general population) rather than lack of availability (25/96, 26.04% vs. 46.4% in the general population). In Africa, lack of interest was more prominent (65/167, 38.92% vs. 26.4%).

Finally, we investigated the differences according to area of living (urban vs. suburban vs. rural) and found that the link between mobility services and OMO scores varied (F(2, 504) = 4.258, *p*=.015, p_(FDR)_=0.045). While it had a significant effect in all area types, a pattern emerged according to which services are more impactful in rural (F(1) = 21.120, *p*<.001) and suburban areas (F(1) = 22.783, *p*<.001) compared to in urban areas (F(1) = 17.451, *p*<.001). As for mobility measures, mobility services seemed to be differently linked to participants’ confidence when travelling in outdoor environments (F(2,504) = 7.155, *p*<.001, p_(FDR)_=0.011): the association was positive in suburban areas (F(1) = 5.509, *p*=.019), null in rural areas (F(1) = 0.152, *p*=.696) and negative in urban areas (F(1) = 11.855, *p*<.001).

### Confirmatory multiple linear regression analysis

We performed the first multiple regression analysis (see Methods) to verify how the provision of mobility services is predicted by the combination of factors such as age, gender, education, economic status, level of functional vision, age at VI diagnosis, WHO region, and area type (rural, suburban, urban). The regression could significantly predict the provision of mobility services (adjusted R^2^=0.195, F(12,451) = 10.353, *p*<.001). Negative associations were found between mobility services and the level of functional vision (B=-0.089, t=-4.014, *p*<.001) as well as with the age of VI diagnosis (B=-0.009, t=-4.384, *p*<.001), confirming that people with lower levels of functional vision and those who received an early diagnosis are more likely to receive these services. Mobility services were also linked to education (B=0.032, t = 2.741, *p*=.006) and age (B=0.005, t = 2.420, *p*=.016). There were also differences according to region of living: with the Americas as the reference, the model shows that individuals in all other world regions, except for Europe, were less likely to receive services (all *p*<.001). More details can be found in supplementary file S3.

Then, we performed multiple regression analyses to establish how mobility services correlated with mobility & independence and well-being measures (see Table 1) when accounting for confounding factors (age, gender, education, economic status, level of functional vision, age at VI diagnosis, WHO region, and area type). The overall regression models could significantly predict all variables except for confidence when traveling in outdoor areas. Within the models for mobility & independence measures, the provision of mobility services emerged as an independent predictor of the frequency of independent travel (*p*=.047), the frequency of travels in familiar (*p*=.010) and unfamiliar areas (*p*=.023), and cognitive mapping skills (*p*=.042), confirming that mobility services is linked to better mobility & independence. Mobility services were also a significant positive predictor of the well-being (*p*<.001) and education level (*p*=.006), but not economic status (*p*=.576). Table 2 displays a summary of each multiple regression analysis performed as well as the regression coefficients associated with mobility services provision.

**Table 2:** Results of multiple linear regression analyses examining the association between mobility services, mobility-related outcomes and social determinants of health

Variable	Regression Model			Association with Mobility Services		
	Adj. *R*²	F	*p*	B	t	*p*
How frequently they travel independently	0.141	6.827	< 0.001	0.264	1.989	0.047
How frequently they travel accompanied	0.087	4.414	< 0.001	0.025	0.247	0.805
For how many reasons do they travel	0.183	7.756	< 0.001	0.206	1.306	0.192
How accessible they perceive their environment	0.238	12.146	< 0.001	0.158	1.785	0.075
How frequently they travel in familiar areas	0.132	6.412	< 0.001	0.286	2.581	0.010
How frequently they travel in unfamiliar areas	0.066	3.505	< 0.001	0.154	2.278	0.023
Confidence when travelling in unfamiliar areas	0.126	6.155	< 0.001	0.106	0.807	0.420
Confidence when travelling in outdoor areas	− 0.013	0.549	0.893	-1.720	− 0.672	0.502
Confidence when travelling in indoor areas	0.169	8.244	< 0.001	− 0.147	− 0.509	0.611
How frequently they require help during travels	0.087	4.404	< 0.001	− 0.100	-1.497	0.135
Cognitive mapping ability score	0.298	16.155	< 0.001	4.256	2.035	0.042
Highest level of education completed	0.175	9.166*	< 0.001	0.509	2.74	0.006
Economic status	0.181	9.503*	< 0.001	0.027	0.56	0.576
Well-being (OMO) score	0.382	23.03	< 0.001	8.66	4.47	< 0.001

For each outcome variable, the table reports the overall fit of the regression model, including the adjusted coefficient of determination (Adjusted R²), F statistic, and associated p-value. The regression coefficient (B), t statistic, and p-value corresponding to the mobility services predictor are also presented. Adj, adjusted; B, beta coefficient. *Models predicting education and economic status were based on F(12,451); all other models were based on F(13,450)

According to the multiple regression analyses described above, region of living was also a significant predictor of many mobility & independence and well-being measures, confirming the previously stated regional differences. Indeed, with the Americas as the reference level, regions where PVI are less likely to receive specialized mobility services (Africa, South-East Asia, Western Pacific) were generally associated with lower social determinants of health and mobility outcomes.

Participants from South-East Asia and Western Pacific were more likely to have lower well-being scores (all *p*<.001) and lower education levels (*p*<.001 and *p*=.033, respectively), while participants from Western Pacific also stated to have a lower economic status (B=-0.226, t=-2.7792, *p*=.005). Participants from South-East Asia and Western Pacific also scored lower in cognitive mapping skills (*p*=.018 and *p*=.020 respectively). Participants from South-East Asia had fewer reasons to travel (B=-1.343, t=-4.248, *p*<.001). Participants from Africa and Western Pacific had lower confidence in unfamiliar areas (*p*<.001 and *p*=.004 respectively). Finally, participants from Africa were more likely to state that walking infrastructure in their region was less accessible (B=-0.705, t=-5.677, *p*<.001) and that they required assistance more often (B=0.28, t = 2.436, *p*=.015).

Nonetheless, the analyses revealed a trend according to which participants from the Americas travel less than other participants. Indeed, compared to those living in the Americas, participants from Africa, South-East Asia and Europe travelled independently more often (all *p*≤.008), those from South-East Asia and Europe travelled accompanied more often (*p*=.001 and *p*=.022 respectively), and those from South-East Asia travelled in unfamiliar areas more often (B=-0.512, t = 3.77, *p*<.001). More details can be found in supplementary file S3.

## Discussion

The goal of this study was to examine how specialized mobility services correlate with independent travel and social determinants of health. Individuals with a wide range of visual impairments and socio-economic and cultural backgrounds responded, revealing several insights into the potential benefits of specialized mobility services. Since service provision varied widely across different global regions, this study allows us to examine the perceived impact for individuals that received these specialized services on mobility and travel, as well as their associations with education, socioeconomic status and well-being.

### Impact of O&M on independent travel

For decades O&M training has been associated with an increased ability to travel independently [31, 56, 57], but scientific literature prior to this study has not thoroughly explored what aspects of travel may be influenced by O&M training and services. Filling this gap, this study draws a clear relationship between independent mobility and O&M services that is consistent across regions of the world and resistant to correction for other confounding factors (i.e., age, education, etc.). The data reflects that individuals who receive O&M services benefit from increased independence and improved cognitive mapping skills, and that they travel more often in familiar and unfamiliar areas outside their home. This suggests that there are substantial gains in autonomy and highlights the potential for reduced burden on caregivers and society once PVI receive O&M services. Furthermore, the present findings also show that, while O&M services are likely beneficial to travel outcomes at all ages, the timeliness of their delivery (developmental context in which visual impairment occurs) may also have a large influence on their impact. Early O&M instruction after VI diagnosis is associated with more confidence and perceived environmental accessibility, particularly in individuals whose blindness occurred early in life. Conversely, later O&M services tended to relate to increased travel in familiar environments, possibly reflecting greater independence acquisition or better adaptation to life changes. Taken together, these results highlight the importance of both early and continued O&M services throughout the lifespan in an individualized manner, offering these services when living or travel situations change to promote the ability for independent travel in new areas. While familiarizing individuals with new environments has been one of the fundamental goals of O&M training since the creation of the field [56], this study provides one of the first confirmatory analysis of this effect, independently from world region.

### Relationship of O&M to social determinants of health and well-being

The present study offers novel insights into the multifaceted impacts of O&M interventions, with ramifications in many life spheres beyond mobility, including positive correlations with higher socioeconomic status, higher levels of education, and higher well-being. This confirmation is of importance, because there is a scarcity of literature relating O&M services to social determinants of health and well-being around the world. Prior to this work, this effect was denoted by a limited set of small-scale studies in different regions of the world. In Saudia Arabia, researchers found a positive impact on several psychological well-being measures, such as interpersonal openness and meaning in life, in female students with blindness and low vision [58]. Another study from Pakistan has shown that O&M services benefit educational outcomes in individuals who received an early diagnosis [47]. Also, O&M services have been demonstrated to be associated with post-schooling employment for young adults in the United States [46], an effect that may be partially attributable to the sense of transportation self-efficacy [22].

Another novel aspect from the present study is that we can analyze these impacts based on the age of onset of the visual impairment or the functional level of vision of participants. While the benefits on educational outcomes was not demonstrated strongly, inequities related to these services and education levels were identified. Interestingly, O&M was more closely correlated with higher education for those with a late VI diagnosis as participants with an early diagnosis of blindness had higher education than those with a late diagnosis overall, a pattern that did not hold true for those with O&M services. Though not directly addressed in this study, several possible reasons may account for this discrepancy. Individuals with early onset VI may have the potential to achieve high levels of education from access to other specialized services, such as braille teachers, or teachers of students with visual impairments (TVI) who primarily focus on educational outcomes [59, 60]. Additionally, during primary schooling, children are not typically independent travelers, which can dissociate, to some degree, school success from mobility skills. Additionally, individuals with late diagnoses may have to search for these services online or navigate complex healthcare systems to be provided access to these services, thus prioritizing access to those with higher levels of education. This phenomenon has been shown in several countries, as PVI or their eyecare providers are often not aware of vision rehabilitation services [61–63].

The same situation arises with socioeconomic status, as those with higher statuses were more likely to receive O&M services in our study. The impact of early intervention for those with early onset could likely improve their socioeconomic status by providing them with more educational and employment opportunities, as demonstrated previously [46]. On the other hand, for those with late onset, O&M may allow them to continue traveling to work and maintain other activities outside the home more efficiently (i.e. shopping) while maintaining employment. Additionally, those with higher socioeconomic statuses may have been more able to afford these services, as they are often not fully subsidized by health care agencies or programs in various countries, such as the United States [64]. Unfortunately, the design of the present study cannot discern whether these patterns may be attributed either to their education or socioeconomic status permitting them access to mobility services or vice versa.

Finally, O&M instruction was significantly correlated, in all categories of respondents, to higher levels of well-being, including in domains such as engagement in activities, connections with others, life space, orientation, and self-determination. However, engagement levels were differently correlated depending on the age of diagnosis and degree of vision loss, as those with early onset and those with functional blindness particularly benefited from O&M in that regard. This highlights the need for early intervention, especially when children are exploring their interests in activities. Together, these results provide strong evidence that PVI feel better about themselves and their position within their community after receiving O&M instruction, supporting the idea that promoting efficient, safe and independent mobility has direct impacts on social integration and success. Based on these findings on the social determinants of health and well-being, we recommend that O&M services should be provided to individuals of all ages with low vision or blindness.

### Variability of O&M service provision across regions

According to the directory of the Academy for certification of Vision Rehabilitation & Education Professionals, more than 3000 COMS are present in 12 countries [65]. However, since many of the practicing professionals are not certified by this agency, the extent of the service provision to adults with low vision and blindness is unknown. The results from this study highlight the lack of access to these services across world regions, but particularly amongst individuals that experience vision loss later in life and live in rural areas, which appears evident independently from WHO regions. Despite this lack of access, O&M services were more strongly linked to well-being in rural and suburban areas, highlighting how important it is to improve access in these areas.

Furthermore, the findings also highlight a disparity in access based on the world regions where they reside, as participants in the WHO South-East Asia and Western Pacific regions were less likely to receive mobility services. To better understand the factors related to these discrepancies in service provision, we explored the reasons behind not receiving services, which appear to reveal differences in social or cultural norms. Responses from the Western Pacific suggest a lack of availability of O&M services, whereas a lack of interest and need for O&M services is substantial in Southeast Asia. Southeast Asia was also one notable exception in regard to the impact of O&M services, which were negatively correlated with perceived accessibility and confidence indoors in participants from that region. This may highlight the extent of the accessibility barriers faced by PVI, which may be understood, but not overcome with current O&M services in this region, as previously highlighted by researchers in India [66].

Based on the inequities of O&M service delivery in the countries studied, we recommend provision regardless of the area where the individual lives or their socioeconomic or educational status. Also, in countries where these services are sparse or lacking should consider efforts to provide specialized training programs to increase the availability of O&M specialists. Since vision rehabilitation requires motivation and engagement on behalf of the client [67] as well as support by family members or caregivers [68], tailored regionally accepted approaches should be adopted to enhance access to O&M services. The stigmatization of PVI in certain regions [7, 69–71] should not be neglected, as this could lead to a lack of interest in receiving these services, as the use of white canes or guide dogs make the disability more visible to the public. Altogether, these results suggest that personal choice should be considered by O&Ms for the type and location of training as well as the orientation and mobility aids to recommend. In relation to this, our prior research highlighted the positive impacts of training the use of tactile maps on cognitive mapping skills and independent travel, especially when taught within O&M services, however the use of these tools remain scarce [12].

### Limitations and future research

Though this study was conducted in four languages, with the support of specialized associations to increase the study’s scope, it is possible that it does not accurately represent the views of those with less technological competency or competency in the language administered. Future research will explore the O&M experiences of PVI from additional countries with translated versions of the questionnaires in four of the other most common languages spoken internationally. Furthermore, this study gathers self-reports (subjective) from PVI regarding their life situation, which could explain, in addition to other potential confounding factors, the moderate to small effect sizes that were observed. The study did not focus on the efficacy of O&M techniques or aids, future research should focus on the types of training and instruction provided by O&Ms in the context of different countries and world regions, as well as their efficacy to promote independent, safe travel. Also, this study did not examine the training received by O&M specialists, thus cannot provide insights into the development of these programs. Future research could explore culturally appropriate training programs to promote these specialized travel and mobility services. Due to the vast diversity in profiles of participants, we cannot pinpoint the exact causes of these disparities between those that received O&M services and those that did not.

## Conclusions

As independent and safe mobility is essential for a wide range of activities, PVI receive substantial benefits from specialized mobility training that extend beyond their direct impact. Our data indicate that increased confidence, more connections with others, the ability to participate in more fulfilling activities, as well as a higher likelihood of having a higher socioeconomic status are tangential benefits to this training. Unfortunately, many individuals still do not receive these services due to a lack of availability in their area. Based on the results of this study, we encourage the expansion of O&M services to individuals with congenital or acquired vision loss of all ages experiencing low vision or blindness that takes into account cultural and societal norms.

## Supplementary Information

Supplementary Material 1.

Supplementary Material 2.

## Data Availability

The datasets generated and/or analyzed during the current study are not publicly available to protect the identity of participants but are available from the corresponding author on reasonable request.

## Abbreviations

CERC: Comité d’éthique à la recherche clinique
COMS: Certified orientation and mobility specialist
EB: Early blind
FDR: Benjamini-Hochberg false discovery rate
LB: Late blind
LV: Low vision
O&M: Orientation and mobility
OMO: Orientation and mobility outcomes
PVI: People with visual impairments
VROOM: Vision-related outcomes in orientation and mobility
WHO: World health organization
