# Supplementary material for: Impact of specialized mobility services on independent travel, economic outcomes, and well-being for people with low vision and blindness: an international survey
Source: BMC Public Health. 2026 Aug 19;26:3372. doi: 10.1186/s12889-026-29083-5 (PMC13760932; doi:10.1186/s12889-026-29083-5)
Supplement: Supplementary file 1 [file 12889_2026_29083_MOESM2_ESM.pdf]

Results

General predictors of OMexperience

Model Summary - OMexperience

Model	R	R^2	Adjusted R^2	RMSE
H_0	0.000	0.000	0.000	0.486
H_1	0.465	0.216	0.195	0.436

ANOVA

Model		Sum of Squares	df	Mean Square	F	p
H_1	Regression	23.593	12	1.966	10.353	< .001
	Residual	85.648	451	0.190		
	Total	109.241	463			

Note. The intercept model is omitted, as no meaningful information can be shown.

Coefficients

Model		Unstandardized	Standard Error	Standardized[a]	t	p
H_0	(Intercept)	0.379	0.023		16.821	< .001
H_1	(Intercept)	0.587	0.132		4.435	< .001
	Education	0.032	0.012	0.126	2.741	0.006
	UtilityVisionGroup	−0.089	0.022	−0.183	−4.014	< .001
	EconomicClass	0.025	0.045	0.026	0.560	0.576
	Age	0.005	0.002	0.133	2.420	0.016
	AgeDiagnosis	−0.009	0.002	−0.234	−4.384	< .001
	WHO_REGION (Africa)	−0.313	0.064		−4.873	< .001
	WHO_REGION (South-East Asia)	−0.448	0.092		−4.861	< .001
	WHO_REGION (Europe)	−0.013	0.095		−0.133	0.895
	WHO_REGION (Western Pacific)	−0.402	0.076		−5.284	< .001
	AreaType (Subur)	0.054	0.070		0.769	0.442
	AreaType (Urb)	0.039	0.062		0.635	0.526
	Gender (M)	−0.066	0.042		−1.559	0.120

[a] Standardized coefficients can only be computed for continuous predictors.

LVorBlind => OMexperience

Model Summary - OMexperience

Model	R	R^2	Adjusted R^2	RMSE
H_0	0.000	0.000	0.000	0.486
H_1	0.461	0.213	0.192	0.437

ANOVA

Model		Sum of Squares	df	Mean Square	F	p
H_1	Regression	23.240	12	1.937	10.156	< .001
	Residual	86.001	451	0.191		
	Total	109.241	463			

Note. The intercept model is omitted, as no meaningful information can be shown.

Coefficients

Model		Unstandardized	Standard Error	Standardized[a]	t	p
H_0	(Intercept)	0.379	0.023		16.821	< .001
H_1	(Intercept)	0.500	0.126		3.970	< .001
	Education	0.031	0.012	0.121	2.625	0.009
	EconomicClass	0.021	0.045	0.022	0.470	0.639
	Age	0.005	0.002	0.141	2.574	0.010
	AgeDiagnosis	−0.009	0.002	−0.242	−4.557	< .001
	WHO_REGION (Africa)	−0.316	0.065		−4.868	< .001
	WHO_REGION (South-East Asia)	−0.462	0.092		−4.994	< .001
	WHO_REGION (Europe)	−0.010	0.095		−0.106	0.915
	WHO_REGION (Western Pacific)	−0.387	0.076		−5.083	< .001
	AreaType (Subur)	0.070	0.071		0.997	0.319
	AreaType (Urb)	0.056	0.062		0.907	0.365
	Gender (M)	−0.071	0.042		−1.666	0.096
	LVorBlind (Low Vision)	−0.169	0.045		−3.767	< .001

[a] Standardized coefficients can only be computed for continuous predictors.

BlindGroup (EB vs LB) => OMexperience

Model Summary - OMexperience

Model	R	R^2	Adjusted R^2	RMSE
H_0	0.000	0.000	0.000	0.494
H_1	0.488	0.238	0.203	0.441

ANOVA

Model		Sum of Squares	df	Mean Square	F	p
H_1	Regression	14.778	11	1.343	6.898	< .001
	Residual	47.324	243	0.195		
	Total	62.102	254			

Note. The intercept model is omitted, as no meaningful information can be shown.

Coefficients

Model		Unstandardized	Standard Error	Standardized[a]	t	p
H_0	(Intercept)	0.420	0.031		13.551	< .001
H_1	(Intercept)	0.666	0.194		3.428	< .001
	Education	0.043	0.017	0.172	2.561	0.011
	EconomicClass	0.056	0.061	0.056	0.918	0.359
	Age	-5.887×10^{-4}	0.002	−0.018	−0.283	0.778
	WHO_REGION (Africa)	−0.416	0.116		−3.587	< .001
	WHO_REGION (South-East Asia)	−0.787	0.148		−5.317	< .001
	WHO_REGION (Europe)	−0.062	0.154		−0.403	0.687
	WHO_REGION (Western Pacific)	−0.535	0.144		−3.709	< .001
	AreaType (Subur)	0.146	0.089		1.631	0.104
	AreaType (Urb)	0.026	0.080		0.326	0.745
	Gender (M)	−0.034	0.059		−0.576	0.565
	BlindGROUP (LB)	−0.138	0.064		−2.166	0.031

[a] Standardized coefficients can only be computed for continuous predictors.

LVGroup (Early LV vs Late LV) => OMexperience

Model Summary - OMexperience

Model	R	R^2	Adjusted R^2	RMSE
H_0	0.000	0.000	0.000	0.457
H_1	0.475	0.226	0.187	0.412

ANOVA

Model		Sum of Squares	df	Mean Square	F	p
H_1	Regression	10.691	11	0.972	5.733	< .001
	Residual	36.620	216	0.170		
	Total	47.311	227			

Note. The intercept model is omitted, as no meaningful information can be shown.

Coefficients

Model		Unstandardized	Standard Error	Standardized[a]	t	p
H_0	(Intercept)	0.294	0.030		9.719	< .001
H_1	(Intercept)	0.245	0.158		1.549	0.123
	Education	0.024	0.017	0.098	1.455	0.147
	EconomicClass	0.019	0.065	0.021	0.297	0.766
	Age	0.005	0.003	0.130	1.803	0.073
	WHO_REGION (Africa)	−0.283	0.081		−3.490	< .001
	WHO_REGION (South-East Asia)	−0.425	0.109		−3.906	< .001
	WHO_REGION (Europe)	0.002	0.118		0.019	0.984
	WHO_REGION (Western Pacific)	−0.327	0.088		−3.728	< .001
	AreaType (Subur)	0.001	0.101		0.010	0.992
	AreaType (Urb)	0.159	0.086		1.845	0.066
	Gender (M)	−0.076	0.057		−1.336	0.183
	LVGroup (LateLV)	−0.165	0.066		−2.517	0.013

[a] Standardized coefficients can only be computed for continuous predictors.

OMexperience => OMO scores (well-being)

Model Summary - OMOscore

Model	R	R^2	Adjusted R^2	RMSE
H_0	0.000	0.000	0.000	22.782
H_1	0.632	0.400	0.382	17.906

ANOVA

Model		Sum of Squares	df	Mean Square	F	p
H_1	Regression	96015.576	13	7385.814	23.034	< .001
	Residual	144288.734	450	320.642		
	Total	240304.310	463			

Note. The intercept model is omitted, as no meaningful information can be shown.

Coefficients

Model		Unstandardized	Standard Error	Standardized[a]	t	p
H_0	(Intercept)	56.940	1.058		53.837	< .001
H_1	(Intercept)	23.387	5.560		4.207	< .001
	Education	1.702	0.486	0.143	3.499	< .001
	UtilityVisionGroup	5.108	0.929	0.223	5.498	< .001
	EconomicClass	8.775	1.860	0.193	4.719	< .001
	Age	0.087	0.078	0.054	1.110	0.268
	AgeDiagnosis	−0.414	0.082	−0.242	−5.071	< .001
	WHO_REGION (Africa)	−0.641	2.711		−0.236	0.813
	WHO_REGION (South-East Asia)	−19.485	3.889		−5.010	< .001
	WHO_REGION (Europe)	−0.510	3.908		−0.130	0.896
	WHO_REGION (Western Pacific)	−15.180	3.222		−4.711	< .001
	AreaType (Subur)	8.518	2.889		2.949	0.003
	AreaType (Urb)	8.079	2.532		3.190	0.002
	Gender (M)	3.396	1.741		1.950	0.052
	OMexperience (1)	8.655	1.935		4.473	< .001

[a] Standardized coefficients can only be computed for continuous predictors.

OMexperience => Education

Model Summary - Education

Model	R	R^2	Adjusted R^2	RMSE
H_0	0.000	0.000	0.000	1.908
H_1	0.443	0.196	0.175	1.733

ANOVA

Model		Sum of Squares	df	Mean Square	F	p
H_1	Regression	330.413	12	27.534	9.166	< .001
	Residual	1354.809	451	3.004		
	Total	1685.222	463			

Note. The intercept model is omitted, as no meaningful information can be shown.

Coefficients

Model		Unstandardized	Standard Error	Standardized[a]	t	p
H_0	(Intercept)	4.334	0.089		48.934	< .001
H_1	(Intercept)	2.157	0.528		4.082	< .001
	UtilityVisionGroup	0.146	0.090	0.076	1.629	0.104
	EconomicClass	0.714	0.177	0.187	4.035	< .001
	Age	0.002	0.008	0.016	0.291	0.771
	AgeDiagnosis	−0.005	0.008	−0.033	−0.605	0.546
	WHO_REGION (Africa)	0.043	0.262		0.163	0.871
	WHO_REGION (South-East Asia)	1.250	0.372		3.362	< .001
	WHO_REGION (Europe)	0.673	0.377		1.785	0.075
	WHO_REGION (Western Pacific)	−0.663	0.310		−2.136	0.033
	AreaType (Subur)	0.529	0.279		1.900	0.058
	AreaType (Urb)	0.681	0.243		2.800	0.005
	Gender (M)	0.171	0.168		1.017	0.310
	OMexperience (1)	0.509	0.186		2.741	0.006

[a] Standardized coefficients can only be computed for continuous predictors.

OMexperience => EconomicStatus

Model Summary - EconomicClass

Model	R	R^2	Adjusted R^2	RMSE
H_0	0.000	0.000	0.000	0.501
H_1	0.449	0.202	0.181	0.453

ANOVA

Model		Sum of Squares	df	Mean Square	F	p
H_1	Regression	23.447	12	1.954	9.503	< .001
	Residual	92.726	451	0.206		
	Total	116.172	463			

Note. The intercept model is omitted, as no meaningful information can be shown.

Coefficients

Model		Unstandardized	Standard Error	Standardized[a]	t	p
H_0	(Intercept)	1.388	0.023		59.685	< .001
H_1	(Intercept)	0.909	0.134		6.775	< .001
	UtilityVisionGroup	0.019	0.024	0.038	0.816	0.415
	Age	0.007	0.002	0.189	3.432	< .001
	AgeDiagnosis	−0.005	0.002	−0.142	−2.610	0.009
	Education	0.049	0.012	0.186	4.035	< .001
	WHO_REGION (Africa)	−0.103	0.068		−1.502	0.134
	WHO_REGION (South-East Asia)	0.163	0.098		1.664	0.097
	WHO_REGION (Europe)	0.193	0.099		1.960	0.051
	WHO_REGION (Western Pacific)	−0.226	0.081		−2.792	0.005
	AreaType (Subur)	0.131	0.073		1.790	0.074
	AreaType (Urb)	0.176	0.064		2.771	0.006
	Gender (M)	−0.038	0.044		−0.855	0.393
	OMexperience (1)	0.027	0.049		0.560	0.576

[a] Standardized coefficients can only be computed for continuous predictors.

OMexperience => TravelIndepedent

Model Summary - FreqTravelindependan

Model	R	R^2	Adjusted R^2	RMSE
H_0	0.000	0.000	0.000	1.326
H_1	0.406	0.165	0.141	1.229

ANOVA

Model		Sum of Squares	df	Mean Square	F	p
H_1	Regression	134.020	13	10.309	6.827	< .001
	Residual	679.558	450	1.510		
	Total	813.578	463			

Note. The intercept model is omitted, as no meaningful information can be shown.

Coefficients

Model		Unstandardized	Standard Error	Standardized[a]	t	p
H_0	(Intercept)	3.470	0.062		56.384	< .001
H_1	(Intercept)	2.047	0.382		5.364	< .001
	Education	0.064	0.033	0.092	1.925	0.055
	UtilityVisionGroup	0.326	0.064	0.245	5.119	< .001
	EconomicClass	−0.179	0.128	−0.067	−1.399	0.163
	Age	0.005	0.005	0.052	0.917	0.360
	AgeDiagnosis	−0.022	0.006	−0.220	−3.913	< .001
	WHO_REGION (Africa)	0.618	0.186		3.323	< .001
	WHO_REGION (South-East Asia)	0.781	0.267		2.927	0.004
	WHO_REGION (Europe)	0.714	0.268		2.662	0.008
	WHO_REGION (Western Pacific)	0.182	0.221		0.822	0.411
	AreaType (Subur)	0.278	0.198		1.400	0.162
	AreaType (Urb)	0.263	0.174		1.513	0.131
	Gender (M)	0.206	0.120		1.727	0.085
	OMexperience (1)	0.264	0.133		1.989	0.047

[a] Standardized coefficients can only be computed for continuous predictors.

OMexperience => TravelAccompany

Model Summary - FreqTravelAccompany

Model	R	R^2	Adjusted R^2	RMSE
H_0	0.000	0.000	0.000	0.990
H_1	0.336	0.113	0.087	0.946

ANOVA

Model		Sum of Squares	df	Mean Square	F	p
H_1	Regression	51.303	13	3.946	4.414	< .001
	Residual	402.350	450	0.894		
	Total	453.653	463			

Note. The intercept model is omitted, as no meaningful information can be shown.

Coefficients

Model		Unstandardized	Standard Error	Standardized[a]	t	p
H_0	(Intercept)	2.571	0.046		55.951	< .001
H_1	(Intercept)	2.108	0.294		7.181	< .001
	Education	0.052	0.026	0.100	2.028	0.043
	UtilityVisionGroup	−0.161	0.049	−0.161	−3.277	0.001
	EconomicClass	0.303	0.098	0.153	3.085	0.002
	Age	−0.011	0.004	−0.153	−2.602	0.010
	AgeDiagnosis	0.008	0.004	0.102	1.761	0.079
	WHO_REGION (Africa)	0.056	0.143		0.390	0.696
	WHO_REGION (South-East Asia)	0.670	0.205		3.264	0.001
	WHO_REGION (Europe)	0.475	0.206		2.300	0.022
	WHO_REGION (Western Pacific)	0.315	0.170		1.854	0.064
	AreaType (Subur)	0.186	0.153		1.220	0.223
	AreaType (Urb)	0.129	0.134		0.966	0.335
	Gender (M)	0.120	0.092		1.305	0.193
	OMexperience (1)	0.025	0.102		0.247	0.805

[a] Standardized coefficients can only be computed for continuous predictors.

OMexperience => nTravelReasons

Model Summary - nTravelReasons

Model	R	R^2	Adjusted R^2	RMSE
H_0	0.000	0.000	0.000	1.588
H_1	0.428	0.183	0.159	1.456

ANOVA

Model		Sum of Squares	df	Mean Square	F	p
H_1	Regression	213.789	13	16.445	7.756	< .001
	Residual	954.157	450	2.120		
	Total	1167.946	463			

Note. The intercept model is omitted, as no meaningful information can be shown.

Coefficients

Model		Unstandardized	Standard Error	Standardized[a]	t	p
H_0	(Intercept)	3.386	0.074		45.919	< .001
H_1	(Intercept)	2.107	0.452		4.661	< .001
	Education	0.146	0.040	0.176	3.701	< .001
	UtilityVisionGroup	0.071	0.076	0.044	0.935	0.350
	EconomicClass	0.425	0.151	0.134	2.809	0.005
	Age	3.239×10^{-4}	0.006	0.003	0.051	0.959
	AgeDiagnosis	−0.018	0.007	−0.150	−2.702	0.007
	WHO_REGION (Africa)	−0.093	0.220		−0.420	0.674
	WHO_REGION (South-East Asia)	−1.343	0.316		−4.248	< .001
	WHO_REGION (Europe)	0.166	0.318		0.522	0.602
	WHO_REGION (Western Pacific)	−0.134	0.262		−0.510	0.610
	AreaType (Subur)	0.035	0.235		0.151	0.880
	AreaType (Urb)	0.457	0.206		2.222	0.027
	Gender (M)	−0.046	0.142		−0.326	0.744
	OMexperience (1)	0.206	0.157		1.306	0.192

[a] Standardized coefficients can only be computed for continuous predictors.

OMexperience => AccessWalkInfrastruc

Model Summary - AccessWalkInfrastruc

Model	R	R^2	Adjusted R^2	RMSE
H_0	0.000	0.000	0.000	0.941
H_1	0.510	0.260	0.238	0.821

ANOVA

Model		Sum of Squares	df	Mean Square	F	p
H_1	Regression	106.415	13	8.186	12.146	< .001
	Residual	303.275	450	0.674		
	Total	409.690	463			

Note. The intercept model is omitted, as no meaningful information can be shown.

Coefficients

Model		Unstandardized	Standard Error	Standardized[a]	t	p
H_0	(Intercept)	2.026	0.044		46.391	< .001
H_1	(Intercept)	1.978	0.255		7.760	< .001
	Education	0.002	0.022	0.004	0.087	0.931
	UtilityVisionGroup	0.226	0.043	0.239	5.310	< .001
	EconomicClass	−0.103	0.085	−0.055	−1.210	0.227
	Age	6.500×10^{-4}	0.004	0.010	0.181	0.856
	AgeDiagnosis	−0.008	0.004	−0.118	−2.221	0.027
	WHO_REGION (Africa)	−0.705	0.124		−5.677	< .001
	WHO_REGION (South-East Asia)	0.026	0.178		0.146	0.884
	WHO_REGION (Europe)	0.191	0.179		1.064	0.288
	WHO_REGION (Western Pacific)	−0.234	0.148		−1.582	0.114
	AreaType (Subur)	0.142	0.132		1.075	0.283
	AreaType (Urb)	0.186	0.116		1.605	0.109
	Gender (M)	0.080	0.080		1.005	0.316
	OMexperience (1)	0.158	0.089		1.785	0.075

[a] Standardized coefficients can only be computed for continuous predictors.

OMexperience => FreqTravelFamiliar

Model Summary - FreqTravelFamiliar

Model	R	R^2	Adjusted R^2	RMSE
H_0	0.000	0.000	0.000	1.100
H_1	0.395	0.156	0.132	1.025

ANOVA

Model		Sum of Squares	df	Mean Square	F	p
H_1	Regression	87.504	13	6.731	6.412	< .001
	Residual	472.358	450	1.050		
	Total	559.862	463			

Note. The intercept model is omitted, as no meaningful information can be shown.

Coefficients

Model		Unstandardized	Standard Error	Standardized[a]	t	p
H_0	(Intercept)	3.483	0.051		68.223	< .001
H_1	(Intercept)	2.492	0.318		7.833	< .001
	Education	0.041	0.028	0.071	1.460	0.145
	UtilityVisionGroup	0.258	0.053	0.234	4.858	< .001
	EconomicClass	0.153	0.106	0.070	1.438	0.151
	Age	−0.006	0.004	−0.082	−1.431	0.153
	AgeDiagnosis	−0.012	0.005	−0.145	−2.567	0.011
	WHO_REGION (Africa)	0.323	0.155		2.081	0.038
	WHO_REGION (South-East Asia)	−0.295	0.223		−1.324	0.186
	WHO_REGION (Europe)	0.340	0.224		1.519	0.129
	WHO_REGION (Western Pacific)	−0.040	0.184		−0.215	0.830
	AreaType (Subur)	0.222	0.165		1.341	0.180
	AreaType (Urb)	0.084	0.145		0.579	0.563
	Gender (M)	0.282	0.100		2.825	0.005
	OMexperience (1)	0.286	0.111		2.581	0.010

[a] Standardized coefficients can only be computed for continuous predictors.

OMexperience => FreqTravelUnfamiliar

Model Summary - FreqTravelUnfamiliar

Model	R	R^2	Adjusted R^2	RMSE
H_0	0.000	0.000	0.000	0.647
H_1	0.303	0.092	0.066	0.626

ANOVA

Model		Sum of Squares	df	Mean Square	F	p
H_1	Regression	17.832	13	1.372	3.505	< .001
	Residual	176.097	450	0.391		
	Total	193.929	463			

Note. The intercept model is omitted, as no meaningful information can be shown.

Coefficients

Model		Unstandardized	Standard Error	Standardized[a]	t	p
H_0	(Intercept)	2.192	0.030		72.951	< .001
H_1	(Intercept)	1.894	0.194		9.751	< .001
	Education	0.002	0.017	0.005	0.108	0.914
	UtilityVisionGroup	0.062	0.032	0.095	1.908	0.057
	EconomicClass	0.106	0.065	0.082	1.628	0.104
	Age	-5.522×10^{-4}	0.003	−0.012	−0.202	0.840
	AgeDiagnosis	−0.002	0.003	−0.033	−0.562	0.575
	WHO_REGION (Africa)	0.118	0.095		1.243	0.215
	WHO_REGION (South-East Asia)	0.512	0.136		3.770	< .001
	WHO_REGION (Europe)	−0.006	0.137		−0.042	0.967
	WHO_REGION (Western Pacific)	0.186	0.113		1.654	0.099
	AreaType (Subur)	−0.117	0.101		−1.160	0.247
	AreaType (Urb)	−0.187	0.088		−2.114	0.035
	Gender (M)	0.022	0.061		0.364	0.716
	OMexperience (1)	0.154	0.068		2.278	0.023

[a] Standardized coefficients can only be computed for continuous predictors.

OMexperience => ConfidenceUnfamiliar

Model Summary - ConfidenceUnfamiliar

Model	R	R^2	Adjusted R^2	RMSE
H_0	0.000	0.000	0.000	1.305
H_1	0.389	0.151	0.126	1.220

ANOVA

Model		Sum of Squares	df	Mean Square	F	p
H_1	Regression	119.098	13	9.161	6.155	< .001
	Residual	669.780	450	1.488		
	Total	788.877	463			

Note. The intercept model is omitted, as no meaningful information can be shown.

Coefficients

Model		Unstandardized	Standard Error	Standardized[a]	t	p
H_0	(Intercept)	2.713	0.061		44.777	< .001
H_1	(Intercept)	2.800	0.379		7.392	< .001
	Education	0.028	0.033	0.041	0.841	0.401
	UtilityVisionGroup	0.205	0.063	0.156	3.240	0.001
	EconomicClass	0.034	0.127	0.013	0.272	0.786
	Age	−0.007	0.005	−0.075	−1.297	0.195
	AgeDiagnosis	−0.009	0.006	−0.089	−1.571	0.117
	WHO_REGION (Africa)	−0.721	0.185		−3.904	< .001
	WHO_REGION (South-East Asia)	−0.057	0.265		−0.215	0.830
	WHO_REGION (Europe)	−0.257	0.266		−0.963	0.336
	WHO_REGION (Western Pacific)	−0.630	0.220		−2.871	0.004
	AreaType (Subur)	−0.103	0.197		−0.524	0.601
	AreaType (Urb)	0.145	0.173		0.842	0.400
	Gender (M)	0.235	0.119		1.978	0.049
	OMexperience (1)	0.106	0.132		0.807	0.420

[a] Standardized coefficients can only be computed for continuous predictors.

OMexperience => OutdoorConf

Model Summary - OutdoorConf

Model	R	R^2	Adjusted R^2	RMSE
H_0	0.000	0.000	0.000	23.529
H_1	0.125	0.016	−0.013	23.680

ANOVA

Model		Sum of Squares	df	Mean Square	F	p
H_1	Regression	4003.235	13	307.941	0.549	0.893
	Residual	252324.524	450	560.721		
	Total	256327.759	463			

Note. The intercept model is omitted, as no meaningful information can be shown.

Coefficients

Model		Unstandardized	Standard Error	Standardized[a]	t	p
H_0	(Intercept)	7.603	1.092		6.960	< .001
H_1	(Intercept)	3.849	7.352		0.524	0.601
	Education	−0.352	0.643	−0.029	−0.548	0.584
	UtilityVisionGroup	0.875	1.229	0.037	0.712	0.477
	EconomicClass	−0.919	2.459	−0.020	−0.374	0.709
	Age	0.013	0.104	0.008	0.130	0.897
	AgeDiagnosis	0.010	0.108	0.006	0.095	0.924
	WHO_REGION (Africa)	3.444	3.585		0.961	0.337
	WHO_REGION (South-East Asia)	1.309	5.143		0.254	0.799
	WHO_REGION (Europe)	0.905	5.168		0.175	0.861
	WHO_REGION (Western Pacific)	−2.356	4.261		−0.553	0.581
	AreaType (Subur)	1.058	3.820		0.277	0.782
	AreaType (Urb)	3.538	3.349		1.057	0.291
	Gender (M)	1.902	2.303		0.826	0.409
	OMexperience (1)	−1.720	2.559		−0.672	0.502

[a] Standardized coefficients can only be computed for continuous predictors.

OMexperience => IndoorConf

Model Summary - IndoorConf

Model	R	R^2	Adjusted R^2	RMSE
H_0	0.000	0.000	0.000	2.926
H_1	0.439	0.192	0.169	2.667

ANOVA

Model		Sum of Squares	df	Mean Square	F	p
H_1	Regression	762.512	13	58.655	8.244	< .001
	Residual	3201.798	450	7.115		
	Total	3964.310	463			

Note. The intercept model is omitted, as no meaningful information can be shown.

Coefficients

Model		Unstandardized	Standard Error	Standardized[a]	t	p
H_0	(Intercept)	6.301	0.136		46.382	< .001
H_1	(Intercept)	4.674	0.828		5.644	< .001
	Education	0.094	0.072	0.061	1.300	0.194
	UtilityVisionGroup	0.302	0.138	0.102	2.179	0.030
	EconomicClass	0.294	0.277	0.050	1.060	0.290
	Age	−0.015	0.012	−0.072	−1.275	0.203
	AgeDiagnosis	−0.006	0.012	−0.029	−0.519	0.604
	WHO_REGION (Africa)	1.079	0.404		2.673	0.008
	WHO_REGION (South-East Asia)	0.642	0.579		1.109	0.268
	WHO_REGION (Europe)	−0.216	0.582		−0.371	0.711
	WHO_REGION (Western Pacific)	−1.910	0.480		−3.978	< .001
	AreaType (Subur)	0.588	0.430		1.366	0.173
	AreaType (Urb)	0.983	0.377		2.607	0.009
	Gender (M)	−0.146	0.259		−0.562	0.574
	OMexperience (1)	−0.147	0.288		−0.509	0.611

[a] Standardized coefficients can only be computed for continuous predictors.

OMexperience => FreqHelp

Model Summary - FreqHelp

Model	R	R^2	Adjusted R^2	RMSE
H_0	0.000	0.000	0.000	0.647
H_1	0.336	0.113	0.087	0.618

ANOVA

Model		Sum of Squares	df	Mean Square	F	p
H_1	Regression	21.882	13	1.683	4.404	< .001
	Residual	171.980	450	0.382		
	Total	193.862	463			

Note. The intercept model is omitted, as no meaningful information can be shown.

Coefficients

Model		Unstandardized	Standard Error	Standardized[a]	t	p
H_0	(Intercept)	2.517	0.030		83.797	< .001
H_1	(Intercept)	2.741	0.192		14.281	< .001
	Education	0.010	0.017	0.030	0.607	0.544
	UtilityVisionGroup	−0.136	0.032	−0.210	−4.252	< .001
	EconomicClass	−0.017	0.064	−0.013	−0.258	0.797
	Age	0.002	0.003	0.043	0.723	0.470
	AgeDiagnosis	0.001	0.003	0.026	0.452	0.651
	WHO_REGION (Africa)	0.228	0.094		2.436	0.015
	WHO_REGION (South-East Asia)	0.232	0.134		1.730	0.084
	WHO_REGION (Europe)	0.027	0.135		0.198	0.843
	WHO_REGION (Western Pacific)	-6.887×10^{-4}	0.111		−0.006	0.995
	AreaType (Subur)	−0.170	0.100		−1.700	0.090
	AreaType (Urb)	−0.228	0.087		−2.611	0.009
	Gender (M)	−0.026	0.060		−0.430	0.667
	OMexperience (1)	−0.100	0.067		−1.497	0.135

[a] Standardized coefficients can only be computed for continuous predictors.

OMexperience => ScoreCognitiveMapSkills

Model Summary - ScoreCognitiveMapSkills

Model	R	R^2	Adjusted R^2	RMSE
H_0	0.000	0.000	0.000	23.114
H_1	0.564	0.318	0.298	19.359

ANOVA

Model		Sum of Squares	df	Mean Square	F	p
H_1	Regression	78706.680	13	6054.360	16.155	< .001
	Residual	168648.869	450	374.775		
	Total	247355.550	463			

Note. The intercept model is omitted, as no meaningful information can be shown.

Coefficients

Model		Unstandardized	Standard Error	Standardized[a]	t	p
H_0	(Intercept)	44.795	1.073		41.747	< .001
H_1	(Intercept)	23.512	6.011		3.912	< .001
	Education	1.551	0.526	0.128	2.950	0.003
	UtilityVisionGroup	9.430	1.004	0.406	9.388	< .001
	EconomicClass	1.782	2.010	0.039	0.886	0.376
	Age	−0.008	0.085	−0.005	−0.100	0.920
	AgeDiagnosis	−0.466	0.088	−0.269	−5.280	< .001
	WHO_REGION (Africa)	−4.343	2.931		−1.482	0.139
	WHO_REGION (South-East Asia)	−10.002	4.205		−2.379	0.018
	WHO_REGION (Europe)	4.209	4.225		0.996	0.320
	WHO_REGION (Western Pacific)	−8.155	3.483		−2.341	0.020
	AreaType (Subur)	2.915	3.123		0.933	0.351
	AreaType (Urb)	3.539	2.738		1.293	0.197
	Gender (M)	2.661	1.883		1.413	0.158
	OMexperience (1)	4.256	2.092		2.035	0.042

[a] Standardized coefficients can only be computed for continuous predictors.
