# Supplementary material for: Impact of specialized mobility services on independent travel, economic outcomes, and well-being for people with low vision and blindness: an international survey
Source: BMC Public Health. 2026 Aug 19;26:3372. doi: 10.1186/s12889-026-29083-5 (PMC13760932; doi:10.1186/s12889-026-29083-5)
Supplement: Supplementary file 2 [file 12889_2026_29083_MOESM1_ESM.docx]

S1. Surveys and Mobility Services Provision Worldwide

S1.1. Surveys 1 & 2

The present study, entitled “Impact of specialized mobility services on independent travel, economic outcomes, and well-being for people with low vision and blindness: an international survey”, is part of a broader project, entitled “The road to independent navigation: an international survey on orientation skills of individuals living with visual impairments.” In this supplementary file, only the questions which rendered data relevant to the present study, its research question and performed analyses are shown. To access the complete surveys, please contact the corresponding author of the published manuscript.

Note: for all questions which rendered ordinal data, the ordered categories (i.e., 1 to 5) are shown in brackets and were not available to participants when answering the surveys.

# 1. SURVEY 1 – Sociodemographics & travel habits

A. To participate in this survey, you must be at least 18 years of age, self-identify as visually impaired (blind, deafblind or low vision). Do you meet these eligibility criteria? Please select only one answer. (Required question)
   - Yes (participant takes survey)
   - No (a thank you message appears)

## SECTION 1. GENERAL INFORMATION

1. How old are you (in numbers)?
2. What gender do you identify to?

- Man
- Woman
- Non-Binary
- I prefer not to respond.

3. In what country do you live?
4. What is your highest level of completed education?

- [1] No education
- [2] Some high school
- [3] High school diploma
- [4] Vocational education (e.g. apprenticeship or trade education)
- [5] Community college diploma or CEGEP diploma
- [6] Undergraduate degree (e.g. Bachelor’s degree)
- [7] Postgraduate degree (e.g. Master’s degree or PhD)

5. What is the name of the condition that caused your vision loss if you know it? *Select all that apply*.

- Albinism
- Cataracts
- Diabetic retinopathy
- Eye(s) removed
- Glaucoma
- Macular degeneration
- Optic nerve hypoplasia
- Retinal detachment
- Retinitis pigmentosa
- Retinopathy of prematurity
- Stargart’s disease
- Usher’s syndrome
- Other (please describe): ___
- I don’t know

6. If applicable, at what age (in numbers) were you diagnosed?
7. Has your vision deteriorated (gotten worse) over time?

- Yes,
- No

8. How would you describe your current visual impairment (in your better eye)?

- I have a central (central vision) visual impairment.
- I have a peripheral (side vision) impairment.
- I have a general visual impairment (both peripheral and central)

9. How would you identify the usefulness of your vision for navigation?

- [1] Vision is useless (I only use non-visual strategies, mostly touch, and sounds)
- [2] Vision is secondary (I use non-visual strategies, but sometimes vision is useful)
- [3] Vision needs backup (I can have some hesitation with vision, I use non-visual strategies to confirm)
- [4] Vision is primary (no touch or aid is needed to confirm vision)

10. [If vision useless] If you identify as functionally blind (no usable vision), at what age (in numbers) did you become functionally blind?
11. In what type of area do you live?

- Urban (city)
- Semi-Urban/suburban
- Rural

12. In which socioeconomic class do you consider yourself?

☐ [3] Upper

☐ [2] Middle

☐ [1] Lower/working

## SECTION 2. TRAVELLING HABITS

1. What is your primary mobility aid?

- Cane
- Guide Dog
- Other: ____

2. What orientation aids do you use to travel? Select all that apply

- Smartphone
- Portable GPS device (example: )
- Visual maps
- Tactile maps
- My memory
- Sighted guide
- Other: ___

3. How many times do you travel independently (not accompanied) outside your home in a week?

- [5] Multiple times a day, everyday
- [4] Once a day,
- [3] Multiple times per week
- [2] Rarely (few times per month or less)
- [1] Never

4. How many times do you travel accompanied (with friend or guide to assist you) outside your home in a week?

- [5] Multiple times a day, everyday
- [4] Once a day,
- [3] Multiple times per week
- [2] Rarely (few times per month or less)
- [1] Never

5. What are the reasons for which you travel outside your home? *Select all that apply*.

- Work
- School
- Groceries (essentials, food, and hygiene products)
- Shopping (in example, clothes or other non-essential products)
- Clinical appointments
- leisure or social events (example: meeting friends/family)
- Exercising
- Other type(s) of activity: ___

6. In the area where you live, how accessible and safe is the infrastructure for pedestrians (e.g., sidewalks, street crossings, traffic control lights, stops). Choose the option that best describe your experience.

- [4] It is very accessible, it is easy to walk around with my level of vision and I feel safe, protected from cars and other vehicles.
- [3] Most places are accessible and safe, but I must avoid some places that are less accessible and safe.
- [2] Accessible and safe pedestrian infrastructure are not common, but I can manage without them, I take precautions to feel as safe as I can be.
- [1] It is not accessible; I do not feel safe when travelling on foot and I avoid it as much as possible.

7. How many times in a week do you travel in familiar areas? (example: your neighborhood, around the workplace)

- [5] Multiple times a day, everyday
- [4] Once a day, everyday
- [3] Multiple times per week
- [2] Rarely (few times per month or less)
- [1] Never

8. How many times in a week do you travel in new unfamiliar/new areas?

- [5] Multiple times a day, everyday
- [4] Once a day, everyday
- [3] Multiple times per week
- [2] Rarely (few times per month or less)
- [1] Never

9. How confident do you feel about travels in unfamiliar areas?

- [1] These trips are very stressful, I avoid them.
- [2] These trips can be stressful, but it is ok when someone is with me.
- [3] These trips can be stressful, but I can manage them on my own.
- [4] These trips are challenging, but not stressful.
- [5] I do not mind travelling in new unfamiliar places.
- [6] I like travelling in new places and find it easy.

## SECTION 3. COGNITIVE MAPPING SKILLS

- *Score on /20*

1. What best describes you in the way you travel outside your home?
   - [0] I rely on others for assistance.
   - [1] I know a few routes, and I stick to them.
   - [2] I know many different routes, but rarely go beyond them.
   - [3] I have a complete mental map of places I know; I can walk around as I please.
2. Regarding the neighborhood where you live, what best describe your level of knowledge about it:

- [0] I do not travel independently in my neighborhood.
- [1] I know some roads and locations but cannot go beyond my regular routes.
- [2] I know some roads and locations, but it is a challenge when I need to deviate from my regular routes.
- [3] I know a lot of different roads and locations; I can adapt to different situations that requires me to deviate from my regular routes.
- [4] I know all the different roads and locations, I can easily walk around, improvise new routes without ever getting lost.

3. Regarding a neighborhood you often go to outside of where you live (example: near the workplace, school, family member’s home), what best describe your level of knowledge about it:

- [0] I do not travel independently around the workplace or school.
- [1] I know some roads and locations but cannot go beyond my regular routes.
- [2] I know some roads and locations, but it is a challenge when I need to deviate from my regular routes.
- [3] I know a lot of different roads and locations; I can adapt to different situations that requires me to deviate from my regular routes.
- [4] I know all the different roads and locations, I can easily walk around, improvise new routes without ever getting lost.

4. How would you rate your confidence in learning the configuration of a new indoor area, for example a shopping mall, workplace or school?

- [0] I am not confident I can travel alone at all.
- [1] I am confident in learning some routes that I will follow everyday.
- [2] I am confident I can learn my way around, but it will take time.
- [3] I am confident that I can learn my way around easily and rapidly.

5. How would you rate your confidence in learning the configuration of a new outdoor area, for example a new neighborhood?

- [0] I am not confident I can travel alone at all.
- [1] I am confident in learning some routes that I will follow everyday.
- [2] I am confident I can learn my way around, but it will take time.
- [3] I am confident that I can learn my way around easily and rapidly.

6. (/3) Let’s say you are walking down one of your daily routes (example, to work, school or family member’s home) and there is construction: the road you usually follow is completely blocked. How hard would it be for you to find another way, and go around it?

- [0] Very hard or impossible
- [1] Possible, but a significant challenge
- [2] It would be a challenge, but nothing I can’t manage.
- [3] Easy

# 2. SURVEY 2 – O&M lessons, strategies, and challenges

## SECTION 1. O&M LESSONS

*An Orientation and Mobility (O&M) instructor provides training that is designed to develop or relearn the skills and concepts needed to travel safely and independently through the environment. O&M training focuses on cues that can be observed by a blind or visually impaired person to allow them to understand their location and how to navigate between familiar locations.*

*An O&M instructor provide services across the life span, teaching infants and children in preschool and school programs, as well as adults in a variety of community-based and rehabilitation settings. O&M services are commonly sought multiple times through the life of a single blind or visually impaired person because they are specific to certain locations, such as homes, workplaces and other necessary destinations that can change throughout life.*

1. **Have you ever had a lesson with a mobility or travel instructor (i.e. Orientation and mobility specialist) similar to what is described above?**

- **Yes**
- **No**

2. What is the professional title of your mobility or travel instructor?

- Orientation and Mobility specialist (certified)
- Orientation and Mobility specialist (not certified)
- Locomotion instructor
- Occupational therapist (OT)
- Teacher
- I do not know.
- Other: ___

3. How do you proceed to get O&M services?

- Call a rehabilitation center
- Call a hospital

- Call a school/centre specialized for people with visual impairments
- Call a governmental association for people with visual impairments
- Call a non-governmental association for people with visual impairments
- Other: ___

4. At what age did you have your first orientation and mobility lesson (in numbers), if known?
5. When was the last time you had such a lesson?

- [1] One month ago, or more recently
- [2] Between one month and six months ago
- [3] Between six months and one year ago
- [4] Between one year and five years ago
- [5] More than five years ago

6. How often do you need to have O&M lessons?

- [5] Often, once a week or more
- [4] At least once a month
- [3] Once every two or three months
- [2] Once every six months
- [1] Once a year

**IF NO IN QUESTION 1:**

7. What is the reason why you never had this type of service?

- It is not available where I live.
- I never had interest in that king of service.
- I don’t feel that I need this type of service.
- I am not ready to have this kind of service.
- Other: ____

## SECTION 2. ORIENTATION STRATEGIES

1. How often do you ask others for help?

- [1] Never
- [2] Occasionally
- [3] Regularly (most times I travel)
- [4] At least once during every trip

## SECTION 3. SCENARIOS

1. Outdoor orientation: you’re in a new city and you get out of a bus, you’re now outside at an unknown intersection (you were not prepared). You want to go somewhere and there is no one there to help you. What do you do to find your way? Please enumerate all your options and tools that you have at your disposal and how they can help you.
   1.1. [1,10] Please rate your confidence in that scenario on a scale from 1 (not confident at all) to 10 (totally confident).
2. Indoor orientation: You enter a new, large, building that you never previously explored (and you were not prepared to). You want to go somewhere inside this building (maybe a room or service) and there is no one there to help you. What do you do to find your way? Please enumerate all your options and tools that you have at your disposal and how they can help you.
   2.1. [1,10] Please rate your confidence in that scenario on a scale from 1 (not confident at all) to 10 (totally confident).

## SECTION 4. OMO TOOL PART B

- Score on /20

*In this section, you will be given statements for five different categories (engagement, connections, life-space, orientation, self-determination). For each category, you will have to choose what statement best corresponds to you and how you are living your life, think about the past month.*

1. Engagement

- [0] I find activities overwhelming or boring.
- [1] My mix of activities is not quite right, but I don’t know how to fix it, or I’m not yet ready for change.
- [2] I like some of my activities, but I’m ready for new directions.
- [3] I’m satisfied with my current mix of activities.
- [4] I find my mix of activities interesting and enriching.

2. Connections

- [0] I am isolated and lonely much of the time; it is hard to connect with others.
- [1] People do things for me, but I have little to offer.
- [2] I know where to go to find people; I link in with people or groups sometimes.
- [3] I meet with people regularly; I feel welcome and included.
- [4] I have mutual friendships; we’re there for each other; I contribute.

3. Life-space

- [0] I’m house-bound; I rarely go beyond the front gate.
- [1] I do routine travel, only in well-known local areas (e.g., home block, local shops)
- [2] I explore in my local community; I like to try different routes.
- [3] I travel to known places beyond the local community (e.g. work, school, visiting friends)
- [4] I like to explore beyond the local community, discovering new places.

4. Orientation

- [0] Even at home, I get disorientated; I have trouble understanding shapes, angles and distances.
- [1] I can find the way at home by myself; beyond home, I need a companion, or I get lost
- [2] I travel alone beyond home; if I get anxious or lost, I rely on help from other people
- [3] I travel alone beyond home; if I get anxious or lost, I can usually work it out by myself.
- [4] I can go anywhere independently; I use mental mapping and I don’t really get lost.

5. Self-determination

- [0] My travel is managed by other people; I don’t make the decisions.
- [1] I need travel restrictions – I’m not always aware of what’s safe and what is not.
- [2] I’m aware of my own limitations, but I limit my travel rather than learning new skills.
- [3] I’m aware of my own limitations; I plan ahead; I get information and help with my travel skills.
- [4] I’m in charge; I evaluate my travel and learn from experience as I go; I develop my own skills.

S1.2. Mobility Services provision across world regions

# Descriptive Statistics

## Descriptives

| WHO_REGION | N | Mean | SD | SE | Coefficient of variation |  |
| --- | --- | --- | --- | --- | --- | --- |
| Africa | 266 | 0.372 | 0.484 | 0.030 | 1.301 |  |
| America | 80 | 0.625 | 0.487 | 0.054 | 0.779 |  |
| Europe | 42 | 0.690 | 0.468 | 0.072 | 0.678 |  |
| South-East Asia | 109 | 0.119 | 0.326 | 0.031 | 2.730 |  |
| Western Pacific | 86 | 0.186 | 0.391 | 0.042 | 2.104 |  |

# Contingency Analysis – Mobility Provision by WHO Region

## Contingency Table

| WHO Region | OM | No OM | Total |
| --- | --- | --- | --- |
| Africa | 167 | 99 | 266 |
| America | 30 | 50 | 80 |
| Europe | 13 | 29 | 42 |
| South-East Asia | 96 | 13 | 109 |
| Western Pacific | 70 | 16 | 86 |
| Total | 376 | 207 | 583 |

Note. Cells report observed counts.

## Chi-Squared Test

| Test | Value | df | p |
| --- | --- | --- | --- |
| χ² | 83.625 | 4 | < .001 |
| N | 583 |  |  |

# ANOVA Results – Mobility Services Provision across world regions (WHO_REGION)

## ANOVA

| Cases | Sum of Squares | df | Mean Square | F | p | η² | η²p |
| --- | --- | --- | --- | --- | --- | --- | --- |
| WHO_REGION | 19.149 | 4 | 4.787 | 24.198 | < .001 | 0.143 | 0.143 |
| Residuals | 114.353 | 578 | 0.198 |  |  |  |  |

Note. Type III Sum of Squares

## Post Hoc Tests

| Comparison | Mean Difference | SE | t | p _(Tukey)_ | P _(Bonferroni)_ |
| --- | --- | --- | --- | --- | --- |
| Africa – America | -0.253 | 0.057 | -4.458 | < .001 | < .001 |
| Africa – Europe | -0.318 | 0.074 | -4.310 | < .001 | < .001 |
| Africa – South-East Asia | 0.253 | 0.051 | 5.000 | < .001 | < .001 |
| Africa – Western Pacific | 0.186 | 0.055 | 3.374 | 0.007 | 0.008 |
| America – Europe | -0.065 | 0.085 | -0.773 | 0.938 | 1.000 |
| America – South-East Asia | 0.506 | 0.065 | 7.723 | < .001 | < .001 |
| America – Western Pacific | 0.439 | 0.069 | 6.353 | < .001 | < .001 |
| Europe – South-East Asia | 0.571 | 0.081 | 7.071 | < .001 | < .001 |
| Europe – Western Pacific | 0.504 | 0.084 | 6.024 | < .001 | < .001 |
| South-East Asia – Western Pacific | -0.067 | 0.064 | -1.041 | 0.836 | < .001 |

Note. P-values adjusted for comparing a family of 5
